# The spatial and temporal reconstruction of a medieval moat ecosystem

**DOI:** 10.1038/s41598-022-24762-w

**Published:** 2022-11-30

**Authors:** Olga Antczak-Orlewska, Daniel Okupny, Andrzej Kruk, Richard Ian Bailey, Mateusz Płóciennik, Jerzy Sikora, Marek Krąpiec, Piotr Kittel

**Affiliations:** 1 Laboratory of Palaeoecology and Archaeobotany, Department of Plant Ecology, Faculty of Biology, University of Gdansk, 59 Wita Stwosza St., 80-308 Gdańsk, Poland; 2 Department of Invertebrate Zoology and Hydrobiology, Faculty of Biology and Environmental Protection, University of Lodz, 12/16 Banacha St., 90-237 Lodz, Poland; 3 Institute of Marine and Environmental Sciences, University of Szczecin, 18 Mickiewicza St., 70-383 Szczecin, Poland; 4 Department of Ecology and Vertebrate Zoology, Faculty of Biology and Environmental Protection, University of Lodz, 12/16 Banacha St., 90-237 Lodz, Poland; 5 Department of Historical Archaeology and Weapon Studies, Institute of Archaeology, University of Lodz, 65 Narutowicza St., 90-131 Lodz, Poland; 6 Faculty of Geology, Geophysics and Environmental Protection, AGH University of Science and Technology, 30 Mickiewicza St., 30-059 Kraków, Poland; 7 Department of Geology and Geomorphology, Faculty of Geographical Sciences, University of Lodz, 88 Narutowicza St., 90-139 Lodz, Poland

**Keywords:** Ecology, Zoology, Ecology, Environmental sciences, Limnology

## Abstract

Moats and other historical water features had great importance for past societies. The functioning of these ecosystems can now only be retrieved through palaeoecological studies. Here we aimed to reconstruct the history of a stronghold’s moat during its period of operation. Our spatio-temporal approach allowed mapping of the habitat changes within a medieval moat for the first time. Using data from four cores of organic deposits taken within the moat system, we describe ecological states of the moat based on subfossil Chironomidae and Ceratopogonidae assemblages. We found that over half (57%) of the identified dipteran taxa were indicative of one of the following ecological states: limnetic conditions with or without periodic water inflow, or marshy conditions. Samples representing conditions unfavourable for aquatic insects were grouped in a separate cluster. Analyses revealed that the spatio-temporal distribution of midge assemblages depended mostly on depth differences and freshwater supply from an artificial channel. Paludification and terrestrialization did not happen simultaneously across the moat system, being greatly influenced by human activity. The results presented here demonstrate the importance of a multi-aspect approach in environmental archaeology, focusing not only on the human environment, but also on the complex ecology of the past ecosystems.

## Introduction

The diversity of habitats and of the mutual connections between organisms and their environment can now be studied in a wide variety of ecosystems, including historical features no longer present. Moats were artificial bodies of water providing defence for inhabited strongholds in medieval times. Ecological studies of moats can therefore provide a window into historical influences of human activity on natural ecosystems, broadening our knowledge of the functioning of such features, of past human societies, and of our coexistence with (seemingly distant) animal communities. Ecological studies can be carried out on moats that have either been preserved from historical times (e.g.^1,2^) or have been recently reconstructed. However, these modern moats do not perform the same function today as in the Middle Ages or Early Modern Period^3^, and their aquatic ecosystems may therefore differ. The medieval stronghold’s inhabitants influenced water conditions by changing its trophic state, creating new habitats by placing construction elements, modifying its hydromorphology and sedimentation of defined types of water deposits, and their enrichment with selected metals (see^4,5^). What were the nature, scale, and consequences of such activities? The only way to get a clear window into those past ecosystems is through palaeoecological studies.

Geochemical studies of historical layers (such as core samples) conducted so far have in many cases confirmed their indicator role in environmental archeology^6^. The clearly higher Pb and Cu concentrations in the sediments of medieval moats have been used to determine the course of historical watercourses. However, the grain and mineral composition of sediments influences their susceptibility to metal sorption^7^. Therefore, geochemical mapping of archaeological sites, aiming to assess the distribution and characteristics of metallic pollutants in the environment, should take into account the location of the studied cores.

The chosen location of cores also influences palaeoecological studies. Typically, palaeoecological studies are based on one core of sediment, taken from the deepest (mostly central) part of the (palaeo)lake, on the assumption that the subfossil remains of the organisms once living in different parts of the water body are passively transported over time and deposited into the deepest area of the lake bottom^8^. This way, representative biotic records can be obtained with only one drilling, reducing cost and time of analyses. However, in the case of macrofossils, diatoms, or cladocerans, littoral taxa can be underrepresented in sequences derived from one core^9–11^. Analogously, chironomid head capsules accumulate mostly near the habitat of the larvae, and offshore transport is primarily observed in the less well preserved early instars^12^, which may produce some biases. Generally, if the subfossil record is expected to adequately represent the environment of sedimentation, and track particular events, multiple coring within the (palaeo)lake or mire is needed^8,13^.

In environmental archaeology, it is recommended to take at least two cores for palaeoecological analyses—one from the studied site (on-site profile) and one from an area separated from direct human activity (off-site) (e.g.^14,15^). This allows examination of both local and regional vegetation history, and also of environmental information, such as past water level or temperature changes. Due to differences in deposition between aquatic and terrestrial systems, “wet sites” or “wet features” (such as a moat) allow examination with use of a wider range of ecological proxy analyses, based on organic deposits within cores sampled directly from archaeological trenches.

Our on-site palaeoecological investigations were an integral part of an archaeological study of a stronghold’s moat system in Rozprza (central Poland; see ‘Study system’ section, below)^5,16^. The main sediment core from the studied moat has already been investigated in detail (see^5^). The results indicated that when the stronghold was inhabited (14th-15th century AD), the moat was infilled with shallow water with possible episodes of rinsing through an artificial channel from the nearby river. Changes in water trophic state were tracked using chironomid-based models, and the Chironomidae-inferred summer temperatures of the Late Vistulian came from the nearby palaeomeander^17^. The Holocene chironomid sequence in the palaeomeander core was too low in abundance for quantitative reconstructions. However, even profiles with a depauperate biotic record can be used to obtain some ecological information, especially if treated simultaneously with other analyses. Therefore, we took additional cores from potentially different habitats (cf.^18^) within the moat, in order to obtain a more complete picture of environmental changes and human impact on the ecosystem in both time and space.

Though chironomids have proven to be useful in environmental archaeology (e.g.^19–23^), they have not previously been used for spatial reconstructions of artificial features. Moats, barays and other anthropogenic ponds have more limited potential to accumulate most of the subfossil remains in one point than lakes, mostly because of the specific basin morphology. Therefore, a carefully constructed spatial approach is required, taking into consideration habitat mosaics within the moat ecosystem. Midge larvae (Diptera: Chironomidae and Ceratopogonidae), especially Chironomidae, represent an ideal proxy for spatial reconstruction of artificial features. They are ecologically diverse, sensitive to environmental changes, and indicative of particular ecosystem conditions and processes^24,25^. Therefore, their distribution in shallow reservoirs with complex morphometry may be diversified and earlier studies based on one sediment profile^5^ need to be complemented in order to cover the spatial aspect.

Our study addresses the general problem of lack of replicability in the environmental sciences. The issue is particularly visible when deriving data from remote places or from the past (e.g. in geology, oceanography, palaeontology, archaeology). In palaeoecology, replicability is applied when studying and comparing the same phenomenon/event (e.g. Younger Dryas) in different sites. However, few studies replicate from the same locality, collecting cores several metres apart. On the few occasions that this small-scale replication has been carried out, important variation has been identified (e.g.^26^).

Considering these issues, our main goal was to reconstruct changes in habitat distribution across the stronghold’s moat system over time. We aimed to examine: (1) the extent to which the Chironomidae and Ceratopogonidae subfossils from the different cores reflected differences in past community structure and environmental conditions in different parts of the moat (spatial aspect), and (2) the extent to which midge communities changed simultaneously and/or evenly throughout the moat basin (temporal/time aspect). We hypothesised that even in such a small and shallow water body as a moat, the differences in midge community structure between cores and time periods would be significant and, thus, the habitat mosaic and other aspects of stronghold functioning could be more fully reconstructed using multiple cores.

## Study site

**Figure 1:**
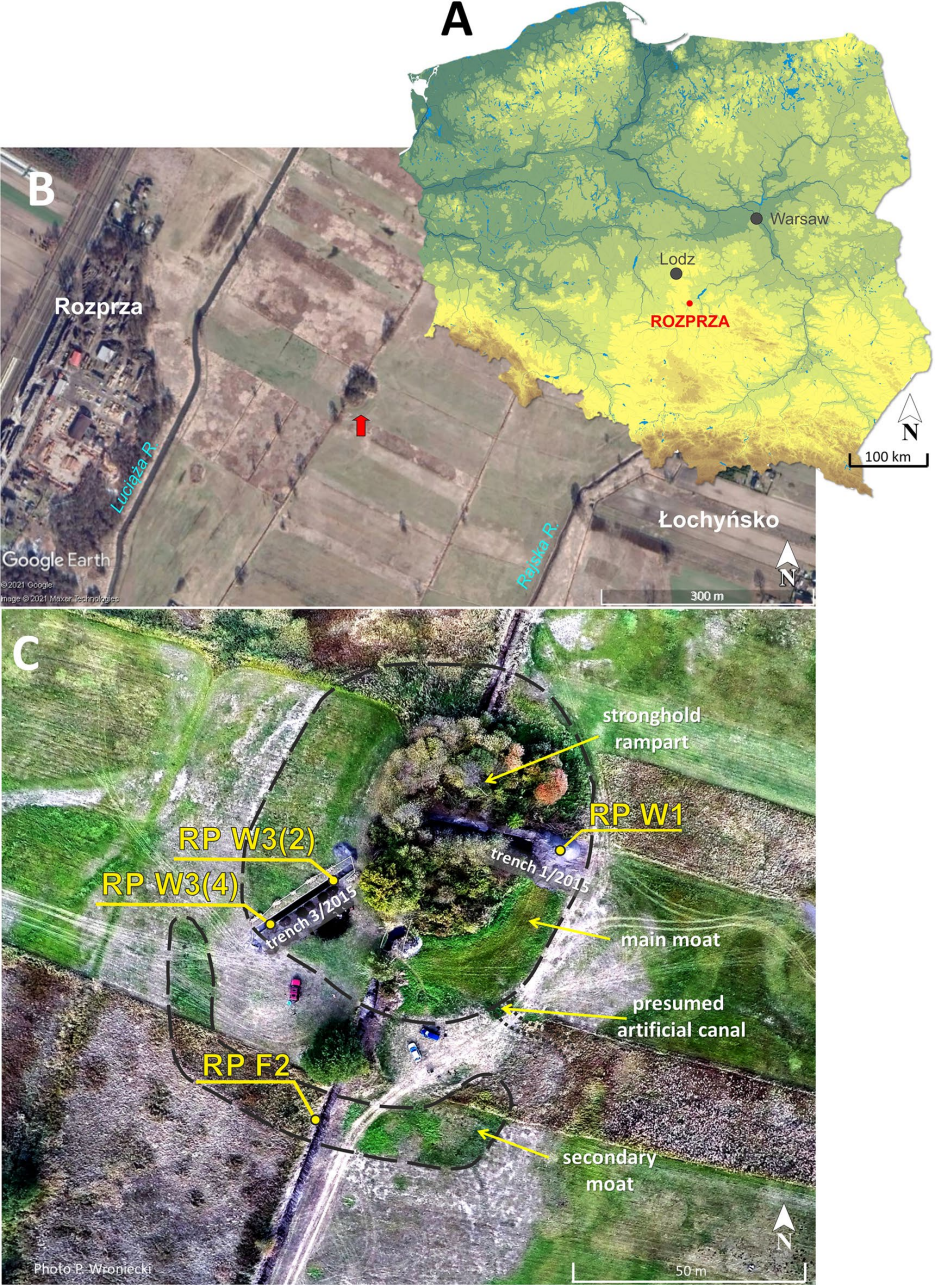
(**A**) Study site location in the territory of Poland. (**B**) The satellite picture of the contemporary surroundings of the study area. The red arrow indicates the stronghold’s remnants (source: Google Earth, modified). (**C**) Aerial photo of the study area. The locations of the studied profiles (cores) are marked by yellow dots. Arrows indicate the traces of artificial elements visible in the terrain relief—moats (dashed lines) and presumed artificial canal (dotted lines) (photo: P. Wroniecki, 2015).

Rozprza is located in central Poland, about 60 km south of Łódź in the Piotrków Plain. The study site is situated in the middle reach of the Luciąża River valley, a tributary of the Pilica River in the Vistula River basin (Fig. 1).

Nowadays, the late medieval stronghold remnants with their moat system are situated in an area covered by fields and meadows between the Rozprza and Łochyńsko villages (51° 18′ 07″ N; 19° 40′ 04″ E). The poorly preserved traces of moats, ramparts and baileys are however still visible in the field (Fig. 1C) and on Digital Elevation Models (DEMs). The study site is located on the valley floor with regulated channels of the Luciąża, Rajska and Bogdanówka rivers, as well as a dense network of drainage canals (Fig. 1B).

The studied fortress was established on the Plenivistulian fluvial terrace remnant, in the area of the widely spread valley floor. Such a location of the motte-and-bailey was reasonable for defensive reasons, as the sandy terrace remnant was protected by the surrounding swampy areas within the valley floor^16,27^. However, the hillock of the terrace remnant occupied by the motte in the late Middle Ages was very low (up to 1 m).

The late medieval motte-and-bailey timber castle at Rozprza was built about 1330 AD and replaced an earlier timber and earth ringfort of unclear chronology (between 11th and 13th century AD). Motte-and-bailey castles were common in western Europe already in the 11th century^28^ but introduced to Poland much later, in the 13th century^29^. In the 14th and 15th centuries AD it was one of the most popular types of rural noble residences.

**Figure 2:**
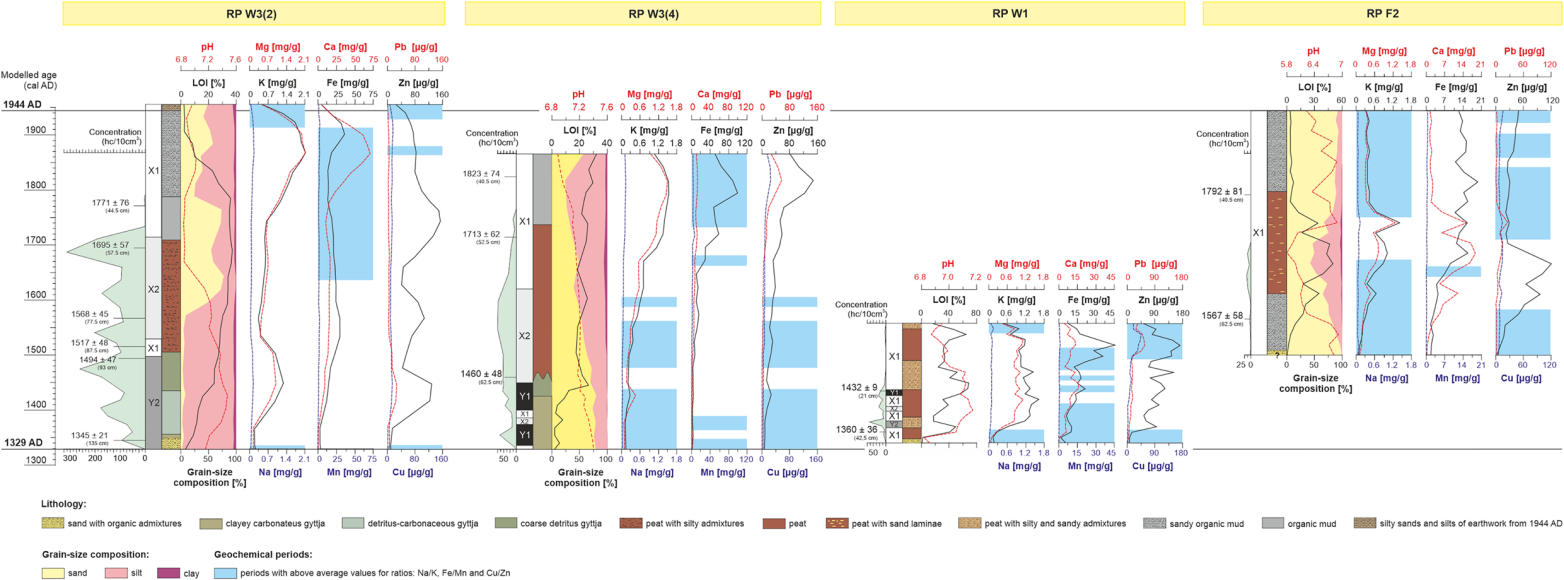
Core correlation with reference to: modelled chronology, midge-inferred SOM subcluster zones (symbols are used as in the Fig. 3), lithology, grain-size composition, lithogeochemical results and statistical relations of selected elements. Midge concentration refers to Chironomidae and Ceratopogonidae head capsules (hc). The data points of the mean age ± 1σ (AD) are given for each core. Question mark means uncertain date of the secondary moat establishment. Geochemical periods were designated on the variable results with respect to average values Na/K (mean = 0.12), Fe/Mn (mean = 60) and Cu/Zn (mean = 0.27) ratios.

The main moat of the Rozprza motte-and-bailey was established ca. 1330 AD and was later filled with organic (gyttja and peat) and partially inorganic deposits containing rich remains of wood (Fig. 2, Supplementary Fig. S1B–D). The fill of the main moat was the subject of a detailed palaeoenvironmental study by Kittel et al.^5^. The accumulation of overbank silty sandy organic mud took place within the moat ditch system as late as in the 18th or 19th c. AD^5,16^. The main moat had a width of 17–21 m and a trapezoidal cross-section with a depth of 0.5 m, up to ca. 1.3 m. Wooden constructions were recorded near the inner slope of the moat ditch—in the form of a palisade created by two rows of vertical, sharpened wooden poles, and horizontal beams lying behind them. Those constructions were covered with thick layers of slope deposits (sand with organic mud). Many large chunks of wood (branches and boughs) were recorded in peat and organic mud of the upper unit of the moat fill close to the inner slope of the main moat^16^.

The second moat had a width of about 11 m and a depth of ca. 0.5 m. In the trapezoid cross-section it has a flat bottom. The fill of the moat consisted of sand with organic admixtures at the very bottom, limnic deposits, peat with silts, sands and organic mud, and also slope wash deposits on the moat slopes. Generally, these sediments are characterised by a massive structure, dark or grey-brown uniform colour, resulting from a strong concentration of dispersed organic matter and iron compounds (Supplementary Fig. S1A). The chronology of the feature formation has been estimated to 1485–1634 AD based on ^14^C data (Table 1). It demonstrates that the secondary moat was built probably in the early 16th century AD. However, the analysis of most archaeological small finds obtained during field works, mostly pottery fragments, estimated their age to the period between 14th and a mid-15th century AD. Evidence of human activity on the stronghold in the 16th century AD is also very limited. Therefore, an establishment of the second moat in the 15th century AD cannot be excluded (see^16^).

**Table 1:** The results of radiocarbon dating of the organic deposits of Rozprza moats.

Core	Lithology	Depth (cm b.g.l.)	Laboratory code/dating method	Dated material	^14^C age (yr BP)	Calibrated age (cal AD)	
						68.2%	95.4%
RP W3(2)	Top of overbank organic mud	42–47	MKL-2839/LSC	Overbank deposits bulk	120 ± 40	1690–1922	1647–1943
	Top of peat	55–60	MKL-2840/LSC	Peat bulk	230 ± 35	1641–1800	1525-…
	Peat	75–80	MKL-2841/LSC	Peat bulk	320 ± 40	1506–1639	1472–1650
	Peat/coarse detritus gyttja	85–90	MKL-2842/LSC	Gyttja bulk	370 ± 35	1458–1622	1449–1635
	Upper part of coarse detritus gyttja	92–94	MKL-3504A/AMS	*Rubus idaeus*—2 seeds *Solanum nigrum*—1 seed	409 ± 36	1440–1615	1428–1629
	Bottom of clayey coarse detritus gyttja	130–135	MKL-2843/LSC	gyttja bulk	1280 ± 50*	667–773*	656–877*
	Sand with plant detritus	134–136	D-AMS 016324/AMS	*Rumex* sp.—2 inflorescences *Chenopodium rubrum*—1 leaf *Urtica dioica*—2 leaves	715 ± 43	1265–1380	1225–1390
RP W3(4)	Top of overbank organic mud	38–43	MKL-2958/LSC	Overbank deposits bulk	100 ± 40	1694–1918	1680–1939
	Peat	50–55	MKL-2957/LSC	Peat bulk	200 ± 40	1655–1950	1642–1950
	Peat/coarse detritus gyttja	60–65	MKL-2956/LSC	Gyttja bulk	460 ± 40	1417–1455	1401–1616
	Bottom of clayey coarse detritus gyttja	75–80	MKL-2955/LSC	Gyttja bulk	1190 ± 60*	710–950*	681–990*
RP W1	Bottom of muddy peat	20–22	MKL-A5577/AMS	*Urtica dioica*—5 fruits *Mentha arvensis*—1 fruit *Thalictrum flavum*—1 fruit *Carex flava*—15 fruits *Sambucus* sp.—1 seed	479 ± 22	1425–1442	1414–1450
	Bottom of peat	40–45	MKL-2609/LSC	Peat bulk	610 ± 50	1305–1397	1289–1415
RP F2	Bottom of sandy organic mud	38–43	MKL-2966/LSC	Organic deposits bulk	80 ± 40	1695–1916	1683–1936
	Bottom of peat	60–65	MKL-2967/LSC	Peat bulk	340 ± 50	1484–1634	1455–1646

*Date recognised as outlier (redeposited material), not included in age-depth model.

## Results and interpretation

### Chronology of moat deposit accumulation

In total six radiocarbon dates were used for the construction of the age-depth model for the RP W3(2) core from the deepest studied part of the main moat (Table 1, Supplementary Table S1)^5,16^. Based on dendrochronological data from a wooden fragment from the moat’s bottom, establishment of the main moat was defined to ca. 1330 AD. In the early phase, the moat was filled with gyttja. In the first half of the 16th century AD, a sedentation of peat began. In the early 18th c. AD the peat was covered with overbank alluvia (Fig. 2).

A comparable pattern of evolution of the main moat was reconstructed based on the age-depth model for the RP W3(4) core (Table 1, Supplementary Table S2). The lacustrine deposition was replaced by peat sedentation in the mid-15th c. AD. The accumulation of overbank alluvia may have been initiated in the first decades of the 18th c. AD. The fill of the western part of the moat was covered in 1944 AD by an embankment from the destroyed stronghold mound^5^.

The chronology of the main moat fills in the RP W1 core confirms an establishment of this moat in the 1st half of the 14th c. AD. Moreover, the eastern part of the wet defensive system was filled with peat from its beginning up to 16th c. AD (Table 1, Supplementary Table S3). The upper part of the moat fill in the RP W1 area was probably removed during melioration works in 20th c. AD.

The absolute chronology of the fill of the secondary moat, studied in the RP F2 core, demonstrates that this additional defensive ditch has been established most probably in the first half of the 16th c. or possibly in the late 15th c. AD (Table 1, Supplementary Table S4). From 18th c. AD, an effect of flooding is visible, recorded by sandy admixtures in organic deposits of the moat fill (cf.^16^).

### Dipterans and environment: identification of the moat phases

The samples from depths of 45-39 cm in RP W3(4) core and the samples from depths of 67 and 49-21 cm in RP F2 core were empty, i.e. without any taxon present. The self-organising map (SOM) allowed clusters of non-empty core samples with similar community composition to be produced. The taxa significantly associated with them were then identified with Indicator Species Analysis.

**Figure 3:**
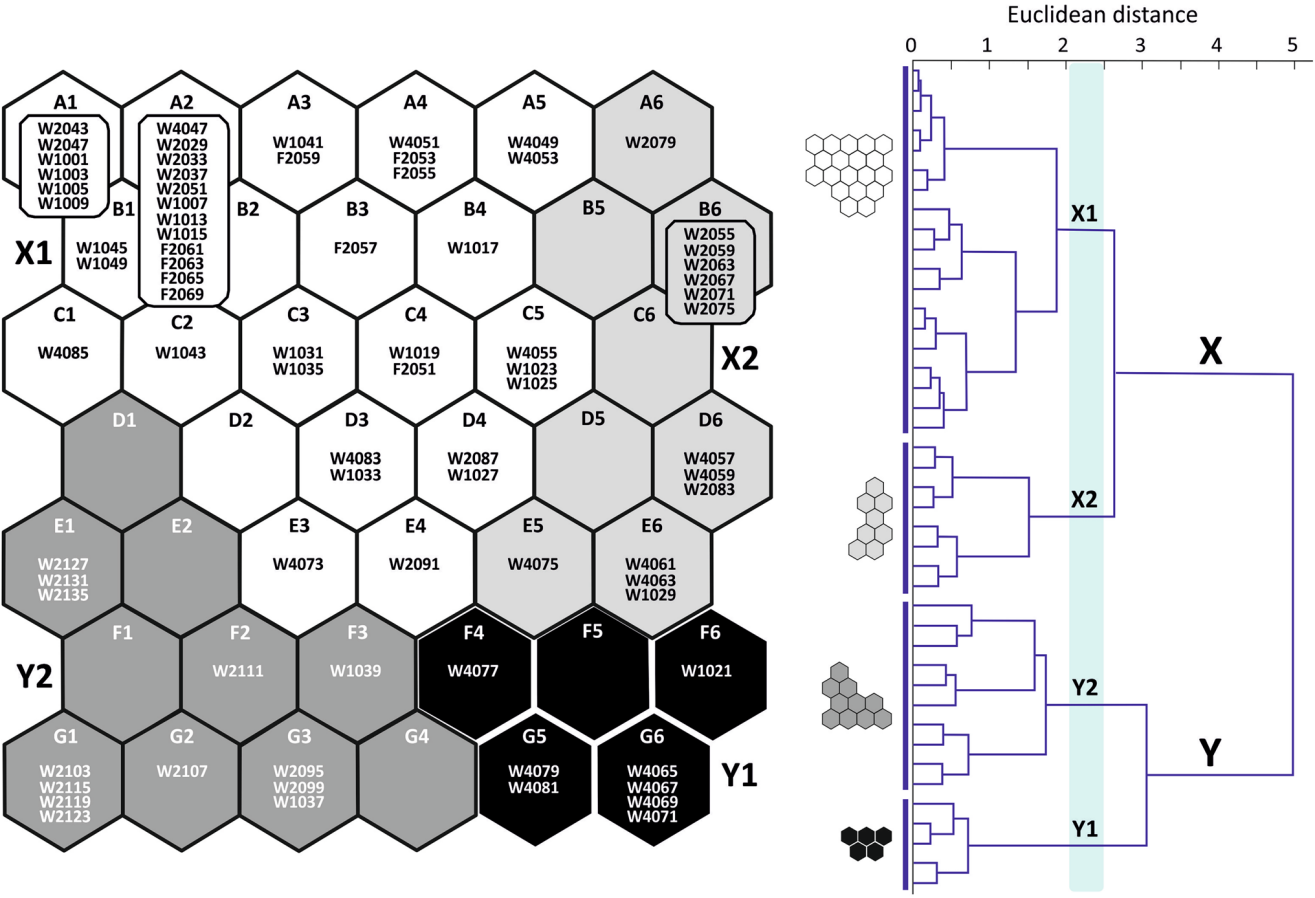
Seventy nine non-empty core samples assigned to 42 self-organising map (SOM) output neurons (A1–G6). The neurons are arranged into a two-dimensional lattice (7 × 6). Clusters (X and Y) and subclusters (X1, X2, Y1 and Y2; shown in different degrees of greyness) of neurons have been identified with the use of hierarchical cluster analysis. Sample codes are arranged as follows: first two signs stand for core symbol (W1—RP W1, W2—RP W3(2), W4—RP W3(4), F2—RP F2), followed by numbers referring to depth (in cm b.g.l.).

**Figure 4:**
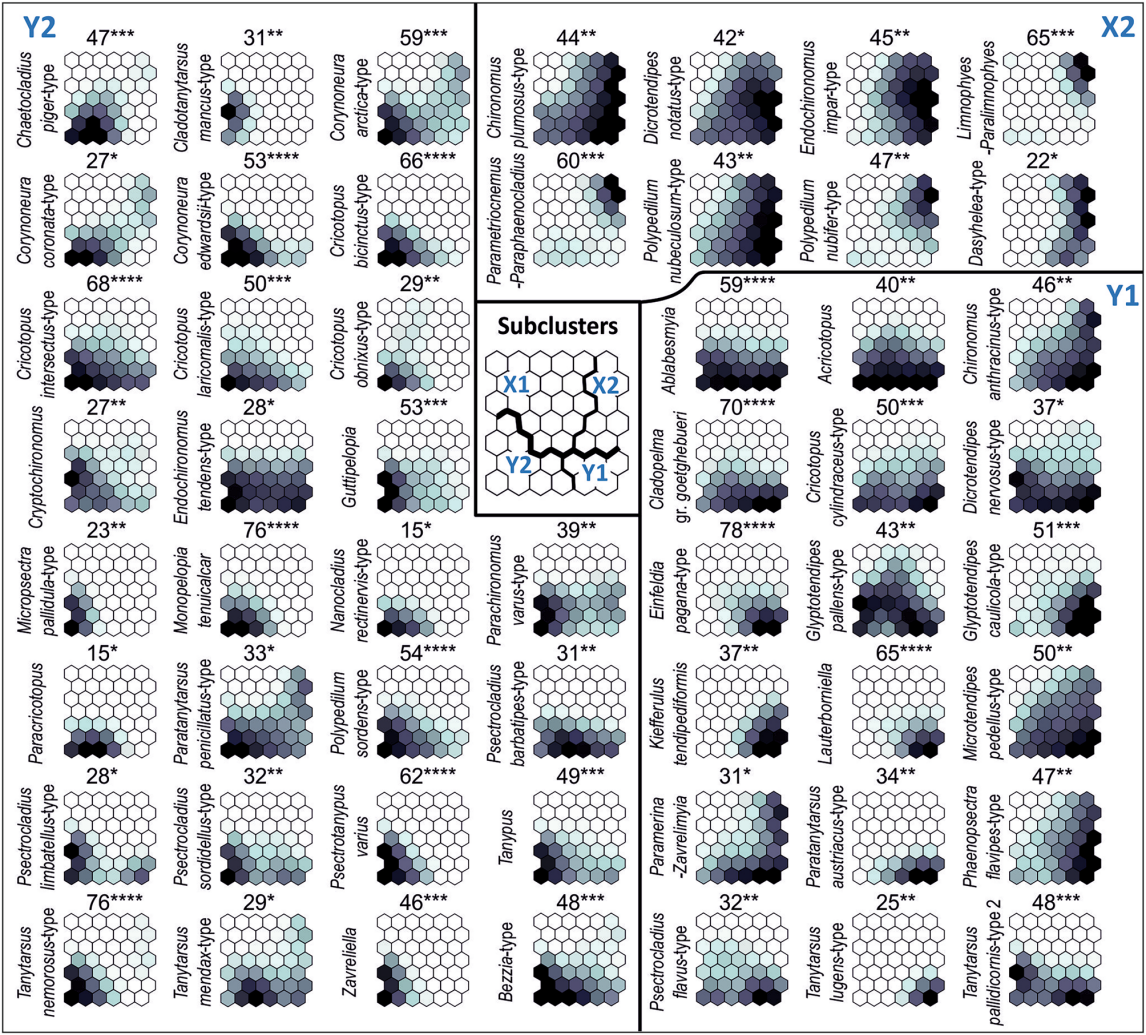
Fifty five dipteran taxa significantly (*p* ≤ 0.05) associated with SOM subclusters X2, Y1 and Y2 (respectively, 8, 19 and 28 taxa). No palaeoindicator was significantly associated with subcluster X1. The shading is scaled independently for each taxon; it is darker for a stronger association in virtual core samples. Maximum observed indicator value (IndVal) is shown above each taxon plane; IndVals and their significance levels were calculated on the basis of real core samples. The plane for *Procladius* (56***), which is indicative of subcluster Y1, is not presented for graphical reasons; it resembles the plane for *Ablabesmyia*.

Two main clusters were distinguished in the output layer of the SOM: X and Y, comprising the respective pairs of subclusters: X1 and X2, and Y1 and Y2 (Fig. 3). The subclusters were ordered according to the gradient observed in the number of indicator species (Fig. 4). Subcluster X1 represents unfavourable conditions for Chironomidae development, mostly overbank deposits (see Fig. 2). It contains surface samples from RP W3 cores (from 51 cm depth in W3(2) and from 55 cm in the W3(4) profile), samples from 91 to 87 cm of W3(2) core depth, 85–83 cm and 73 cm of W3(4) core depth, most samples from RP W1 core, and the whole sequence of the non-empty samples from the second moat (RP F2 core). Subcluster X2 included samples from 83 to 55 cm of RP W3(2) core, 75 cm and 63–57 cm of RP W3(4) core, as well as one sample (29 cm depth) from RP W1 core. They were associated with high organic matter (OM) content (mean 59.9%) and slightly acidic (mean pH = 6.6), probably telmatic (marshy) conditions (Fig. 2). Cluster Y represents limnetic conditions dominated by fully aquatic midges and low values (i.e. < 20) of TOC/N ratio for deposits. The latter suggests that organic matter mainly came from algal phytoplankton^49,81^. Subcluster Y2 reflects habitat with higher detrital (K, Mg, Ca) and sulphide (Cu, Fe) element concentrations, grouping bottom samples from the RP W3(2) core up to 95 cm and samples of 39–37 cm depth from the RP W1 core (Fig. 3). Samples grouped in the subcluster Y1 (81–77 cm and 71–65 cm core RP W3(4) depth, sample from 21 cm core RP W1 depth) are associated with lower sulphide element content (Fig. 3).

A total of 55 (57%) dipteran taxa were significantly associated with a certain subcluster, i.e. they were indicators of its respective environmental conditions (Fig. 4). Among these, 24 exhibited IndVals significant at *p* ≤ 0.001, 20 at *p* ≤ 0.01, and 11 at *p* ≤ 0.05. An upward trend was observed in the number of such taxa for subclusters in the order X1, X2, Y1, Y2. No palaeoindicator was significantly associated with subcluster X1, eight taxa were significantly associated with X2, 19 with Y1 and 28 with Y2. Therefore, this order of subclusters corresponds to increasingly favourable conditions for development of a rich biota.

The most indicative (at *p* < 0.001) morphotypes for subcluster X2 were *Limnophyes-Paralimnophyes* and *Parametriocnemus-Paraphaenocladius*, which are associated with the semi terrestrial habitats with slightly acidic water^30^. Ceratopogonid species grouped as *Dasyhelea*-type seem to have similar preferences^31^, while Chironomini taxa linked to X2 prefer shallow, muddy water bodies and can occur in seasonal surface water. Chironomids associated with Y1 were mostly typical of warm, productive, littoral habitats, and many of them are phytophilous (e.g. *Glyptotendipes pallens-*type, *Lauterborniella*). However, also associated with this subcluster were *Tanytarsus lugens*-type and *Paratanytarsus austriacus*-type, often recorded in cold, oligotrophic conditions. Morphotypes significant to subcluster Y2 included both taxa associated with warm, eutrophic stagnant water (e.g. *Micropsectra pallidula*-type, *Cladotanytarsus mancus*-type, *Cryptochironomus*) and those preferring meso- and oligotrophic conditions (e.g. *Psectrocladius barbatipes*-type and *Bezzia*-type). Many of them, such as *Zavreliella* and *Polypedilum sordens*-type are associated with macrophytes. Moreover, several chironomids associated with running water (such as *Nanocladius rectinervis*-type, *Corynoneura coronata*-type and *Psectrotanypus varius*) were recorded with a significant IndVal in this subcluster. This differentiation is confirmed by the results of the chemical composition filling from the upper part of the RP W3(2) core (Fig. 2), because the rich organic sediments (OM even above 90%) are covered by acidic deposits with organic matter content below 7% and very variable concentration of lithophilic elements (for example K range 0.17–2 mg/g). The changes in time of sorption capacity were probably caused by changes in the porosity of the sediments that accumulated in the moat. This feature is the result of the difference between natural and dry bulk density and it is particularly modified by the content of very fine fraction in the sediments^32^. In turn, the increased abundance in nutrients results from a high proportion of the clay fraction, which in the sediments from RP W3(2) often exceeds 3%, with a maximum of 6.45% (Fig. 2). These features of the biogenic accumulation environment influenced conditions for the development of vegetation and chironomids.

**Figure 5:**
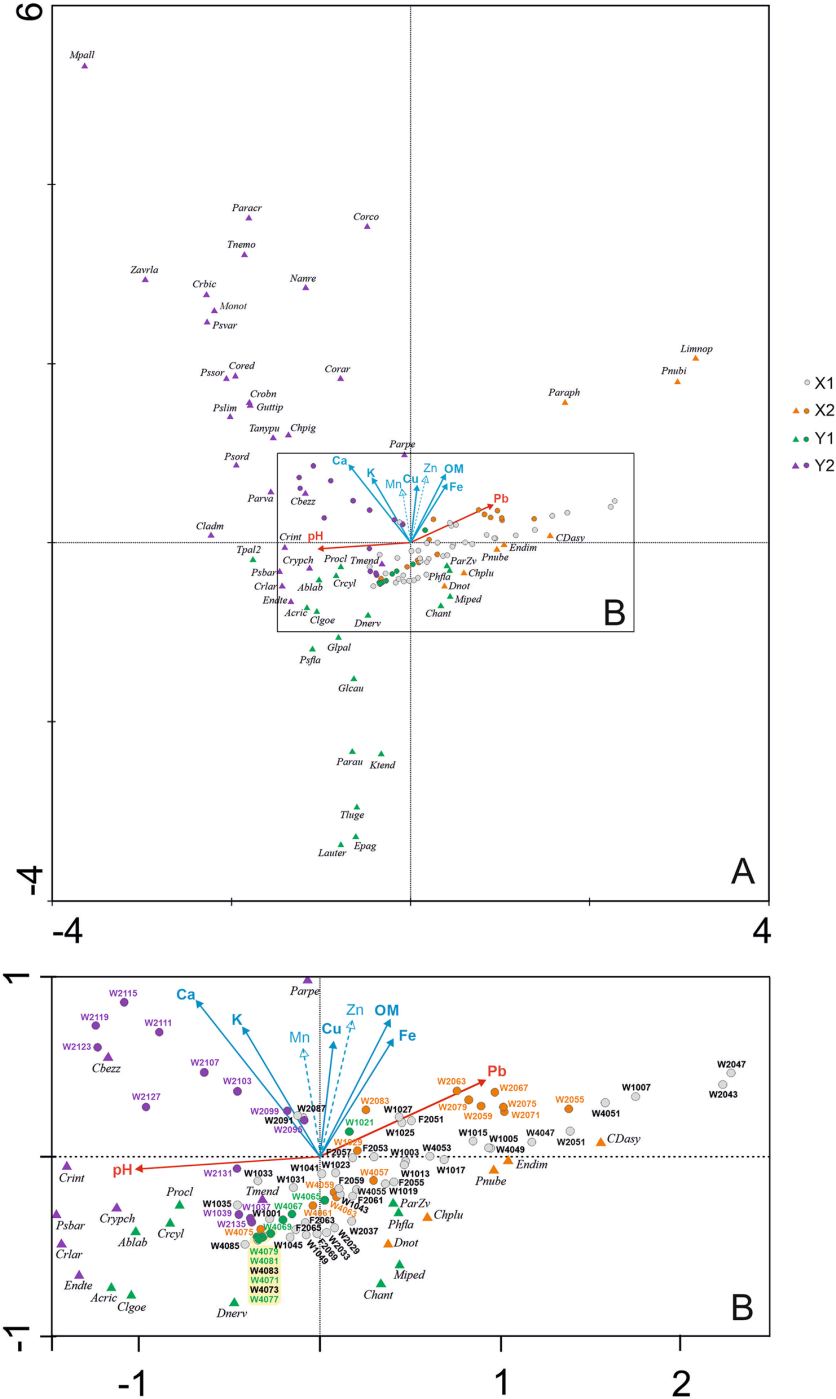
CCA biplot showing changes in the moat states expressed by SOM subclusters represented by indicative dipteran taxa (triangles) and sediment samples (circles), under a gradient of environmental variables (**A**). Variables correlated with Ax1 are shown as red arrows, while those correlated with Ax2 are shown as blue arrows. Mn and Zn were not significant for the analysis. Taxa and samples associated with each subcluster are coloured differently. Sample codes given on zoom (**B**) are arranged as follows: first two signs stand for core symbol (W1—RP W1, W2—RP W3(2), W4—RP W3(4), F2—RP F2), followed by numbers referring to depth (in cm b.g.l.). For full names of taxa see Supplementary Table S5.

Canonical correspondence analysis (CCA) was carried out to detect midge (Chironomidae and Ceratopogonidae) geochemical signal correlations. Axis 1 (Ax1) explained 11.3% and Axis 2 (Ax2) 4.2% of species data variance among individual core samples. For the species-environment relationship variance, 42.1% was explained by Ax1 and 16.0% by Ax2. The analysis (Fig. 5) demonstrated that pH, Ca, Pb, Fe and organic matter (*p* < 0.01), as well as Cu and K (*p* < 0.05), were significant in shaping midge assemblages in the moat, with 6.4% of the variance explained by pH, 4.6% by Ca, 4.2% by Pb, and 3.0% by Fe. Organic matter (OM) and Cu both explained 2.3%, while K explained 1.5% of the total variance. Pb was positively correlated and pH negatively correlated with Ax1. The rest of the variables were positively correlated with Ax2.

The samples grouped in the X1 subcluster generally represented conditions unfavourable for aquatic biota. Many of them were characterised by relatively high Pb, probably reflecting increased denudation processes after stronghold abandonment and increased flooding activity in the 18th–19th centuries AD (cf.^5^). This series describes monofraction of the mineral admixture (share of the sand mainly ranges between 70–90% and M_z_ for 70% number of samples is 1.6–2.2 *phi;* Fig. 2). According to Kittel et al.^21^, in the absence of a clear boundary between individual layers, identification of flood activity should include changes of colour sediments, caused by admixture of decomposed and diffused organic matter (Supplementary Fig. S1). In our case, organic matter values varied little among X1 subcluster samples (mean for this section 29.3%) and corresponded with light-grey horizon (overbank organic mud and overbank sandy organic mud *vide*:^5^; Fig. 2) a dozen cm thick. The overbank deposits accumulated in rapidly changing oxygenation of water. Such conditions stimulated microbial decomposition of organic matter and allowed accumulation of sulphide elements, such as Cu, Mn and Fe (Fig. 2). The taxa typical of the telmatic phase of the moat (X2) were associated with low pH. Moreover, *Limnophyes-Paralimnophyes, Parametriocnemus-Paraphaenocladius* and *Polypedilum sordens*-type prefer habitats with high organic matter and iron compounds content. However, among samples classified to the X2 cluster, those from RP W3(2) core were more related to acidic conditions than those from RP W3(4). Generally more alkaline conditions are preferred by the chironomids indicative of the limnetic stage of the moat, in particular to subcluster Y2 (Fig. 5). Those taxa (e.g. *Zavreliella* and *Cricotopus bicinctus*-type) prefer habitats with high Ca and K values. Several phytophilous taxa (such as *Paratanytarsus penicillatus*-type, *Corynoneura coronata*-type and *C. arctica*-type) seemed to be more associated with sulphide elements (Cu, Zn), than with pH level. Alkaline conditions are also important to several taxa indicative of the Y1 subcluster (like *Tanytarsus pallidicornis*-type 2), but unlike Y2, these taxa are associated with low element levels (Fig. 5). The occurrence of allochthonic mineral matter with variable grain-size parameters (Supplementary Fig. S2) in the Y1 samples may be responsible for the increased lithophilic elements (mainly Mg and K) content in deposits (Fig. 2).

The high concentrations of Ca (often above 60 mg/g) (Fig. 2) are documented in the deposition environment of the hypergenic zone in Central Europe (cf.^33^). The intensive chemical denudation and leaching of mineral substrate of variable origins were confirmed in the catchment of Luciąża River valley, the surface geology and mineralogy of which was documented in detail by Wachecka-Kotkowska^34^. Moreover, the documented sediment structures and textures (Fig. 2, Supplementary Figs. S1 and S2) prove the supply of mineral matter in two different accumulation environments. The first one is related to upper flow regime (mainly samples from RP F2 and RP W3(4)), the second one in the lower flow regime conditions, which is related to the sorting by absorbing detrital material by selective deposition of fine grains from the suspension^35^.

In most of the studied profiles the concentration of sulphide elements was low (Cu: 3.49–57.2 μg/g; Zn: 3.12–210 μg/g; Fe: 1.2–99.3 mg/g) (Fig. 2), but irrespective of lithology, these results are typical for a river valley environment in Central Europe^36^. The stratigraphy differentiation in deposit chemistry indicated that enrichment of Cu and Fe took place during the changes of sedimentation type from organic rich to mineral input or increased humification. Precipitation of colloidal forms of these elements was dependent on changes in the local groundwater level^37^. In the Luciąża River valley the water budget was represented by water flowing underground into the alluvia coming from the post-glacial areas surrounding the Rozprza stronghold (e.g. Radomsko and Dobryszyce Hills), the water of the Luciąża river system, and precipitation water that did not participate in the evapotranspiration processes.

### Testing differences among cores in relative taxon abundance

To examine whether an individual core can be considered representative of the whole site, we tested for differences in relative dipteran taxon abundances among cores, controlling for sample volume and sample age effects. Diagnostics revealed no significant deviations from model assumptions for the fitted poisson family generalised linear mixed model (GLMM). Fixed effect model selection based on AICc revealed the full model to be the best-fitting model, including an interaction between core and species (LL = − 3727.7, d.f. = 294, AICc = 8079.1, weight = 1). This indicated a significant difference among cores RP W1, RP W3(2) and RP W3(4) in chironomid relative species abundance distributions. Therefore, there may be some error associated with extrapolating results from a single core across a whole site. The species with the biggest differences among cores in relative abundance were *Chironomus plumosus*-type and *Dicrotendipes notatus*-type, which both are common, often with high share in the samples. However, they had much higher relative abundance in core RP W3(2) than RP W1 (Supplementary Table S5).

## Discussion and conclusions

The results based on chironomid and ceratopogonid assemblages generally confirm three main stages of the moat history: limnetic, telmatic and terrestrial. In addition to the previous study^5^, however, we reveal variation in moat habitat overall and in temporal habitat changes across the moat system.

**Figure 6:**
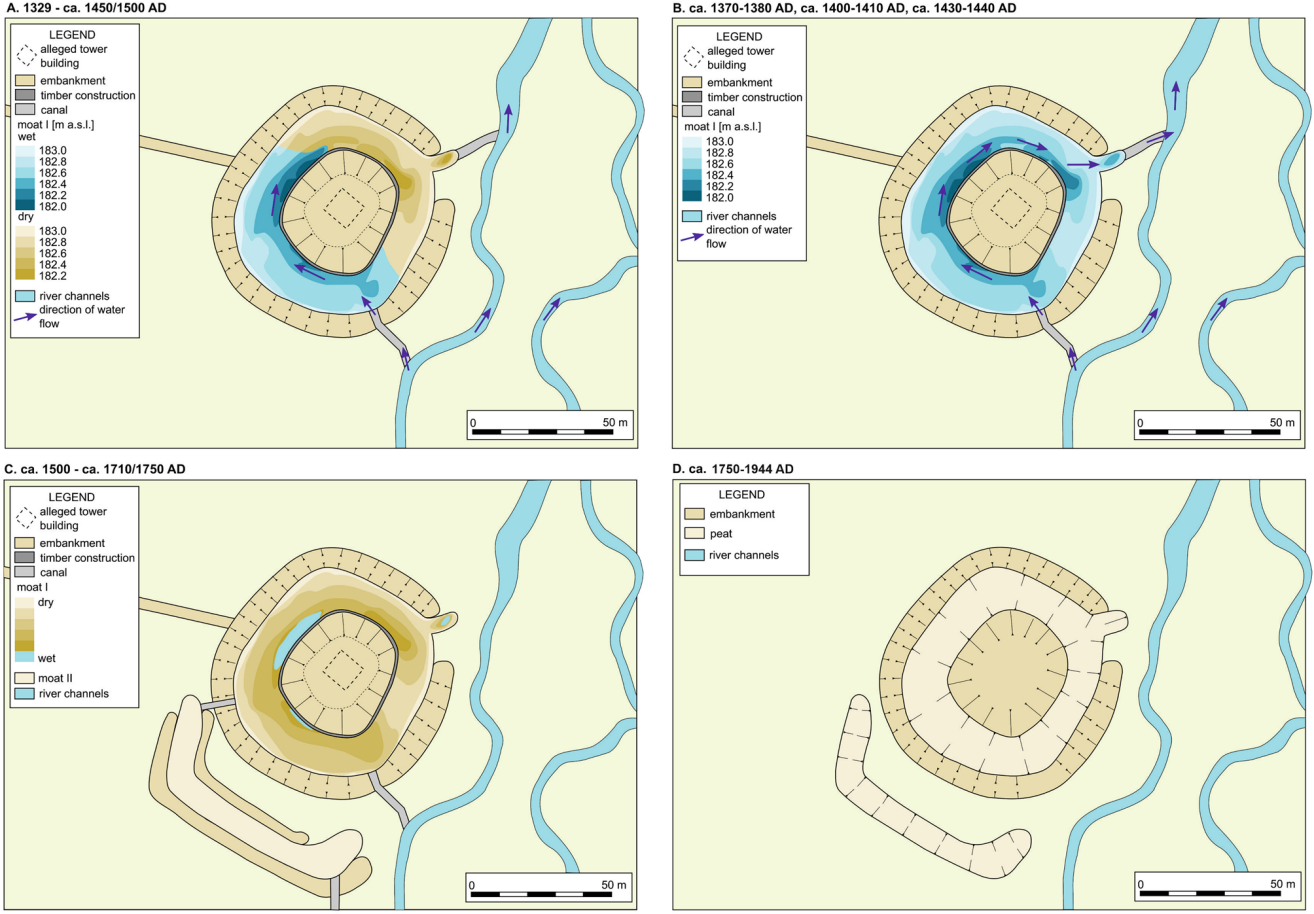
Phases and episodes of different moat states over time: (**A**) limnetic stage only in the southern (deeper) part of the main moat (1329—ca. 1450/1500 AD); (**B**) – limnetic stage in the whole main moat, episodes of higher water level in the NE part of the moat, indicated by Y2, X2 and Y1 subclusters in the core RP W1 (ca. 1370–1380 AD, ca. 1400–1410 AD, ca. 1430–1440 AD); (**C**) construction of the southern secondary moat, telmatic stage (ca. 1500—ca. 1710/1750 AD); (**D**) terrestrial stage in the both moats up to their covering with the material from the stronghold’s mound in 20th century AD (ca. 1750–1944 AD).

The afore-mentioned three stages of the moat are visible only in its deepest, south-western part (both RP W3 cores) (Fig. 2). Here the ecological processes, such as paludification, lasted longer and the habitat changes were less dynamic, resulting in more stable conditions for biota. However, the limnetic phase was of a different nature in the RP W3(2) and RP W3(4) cores, which were located close (12.5 m) to each other. While fresh water from the artificial canal (see Figs. 1C, 6,^5^) firstly supplied the southern part of the moat, the inflow may have been higher in RP W3(2) than in RP W3(4), probably because of its greater depth (Fig. 6). The significant presence of rheophilic taxa in the former core (indicative for cluster Y2) supports this. Moreover, the sediment chemistry record (Fig. 2), in particular values of Fe/Mn ratio, suggest higher oxygenation in this part of the moat, which may indicate the course of the water current. A crucial factor in this case could also be the structure of the moat bottom closely related to the groundwater level, which determines the habitat diversification of the plant cover. These processes could lead to the aggregation of soils grains/sediment into concretions and lumps, which when combined with Fe and decomposed organic matter, can lead to development of dense hardly permeable zones^38^. Such a situation in the studied area would have a direct impact on the disturbance of vertical water movement and the possibility of plant rooting, determining the specific geochemical cycle between moats-plants-sediments. While habitats in both RP W3 cores during the limnetic phase, with high pH and dense vegetation, could support well-functioning biotic communities, conditions in the shallower part of RP W3(4) were slightly less favourable for midges. There, the limnetic stage is reflected mainly in the Y1 subcluster, interrupted by single samples with lower concentration of midge larvae. The telmatic phase in this core could have started earlier than in RP W3(2), as indicated by peat deposits and the X2 cluster. This effect may be caused by the location of RP W3(4) close to the moat edge, resulting in faster sedentation and shallowing of the moat bottom.

The north-eastern part of the main moat had worse conditions for chironomid development, as is shown by cluster X1 containing most samples from the RP W1 core (Fig. 2). Generally, the RP W1 core is characterised by relatively uniform lithology, consisting mostly of peat (with periodical supply of the mineral fraction, including sands). It is the result of much shallower conditions in this part of the main moat resulting in dominating sedentation of peat. During the first phase of the moat history (up to ca. 1440 AD), episodes of more complex midge larvae assemblages were recorded, as reflected by clusters Y2, X2 and Y1 (Fig. 2), and higher chironomid richness and abundance (Supplementary Fig. S5). They may indicate some limnetic episodes also in the NE part of the moat (Fig. 6), though not as clear and stable as in its deeper southern part. Despite slight differences in the concentration of most elements, they are confirmed by selected geochemical indicators (e.g. increase of Fe/Mn ratio to 82). These episodes are also accompanied by a clear decrease in the values of denudation indicators (i.e. Na/K from 0.12 to 0.04 and Ca/Mg from 0.03 to 0.01) (Fig. 2). The record of the RP W1 core ends approx. 1560 AD, probably because of the anthropogenic removal of the top parts of the moat filling during drainage works in the 20th c. AD.

Generally, the history of the main moat lasted for six centuries, since its establishment in ca. 1329 AD till the earthwork from 1944 AD, when the moat remnants were levelled (Fig. 6). In fact, the moat was only functioning as a defensive water body for the first 120–170 years of its existence. Even then it was fully aquatic only in its deepest, south-western part, with only several recorded episodes of higher water level in the whole feature (Fig. 6B). Its status changed in the 15th century, when it dried up (or was drained), becoming a kind of wetland, and ca. 300 years later it was fully filled with organic deposits and covered by overbank mineral matter.

The second moat (RP F2 core) was functioning briefly, as it was created not earlier than in the 2nd half of 15th century, and most probably ca. 1500 AD (see Fig. 2, Supplementary Table S4). In fact, it is not certain whether it was built as a functional moat, or possibly as a dry ditch (e.g. for melioration or defensive purposes). The chironomid scarcity and high admixture of sands (Figs. 2, 5) rather support the latter possibility. Moreover, the secondary moat was much narrower (ca. 11 m) than the main one, so active slope processes provided a constant supply of mineral matter (mostly sands).

The results of habitat reconstruction confirm the modelling (GLMM) outcome that one core does not show the entire history of the moat. This is because a moat is not a typical water body—not only very small and shallow, but also with a specific shape, which hinders transport of fossils to the deepest part of the moat. The steep, almost vertical banks, artificial channels, depth differences and many other features had a crucial impact on the sediment spatial composition and, hence, also on moat functioning. Anthropogenic wood and other artefacts and ecofacts in the bottom can serve as an additional habitat, e.g. for periphyton development.

Besides Rozprza, only a few moats in Europe had hitherto been studied using palaeoecological analyses (e.g.^39–41^), and they mostly focused on human economy and functioning, rarely touching the issue of the moat ecology itself. Moreover, some of these studied defensive objects were dry (e.g. in Prague^42^ and Gdańsk^43^). The external moat of the Czermno stronghold^44^ seems to be comparable with the Rozprza site, though they cover different time spans. Both features evidenced relatively fast peat sedentation and paludification in comparison with natural water bodies (cf.^17^). The moat system in the Tum (Łęczyca) stronghold has been well studied, including palynological and plant macrofossil analyses from its different parts^45,46^. However, no further spatial reconstruction of the environmental conditions within the feature was provided.

Moats and similar human-made features are hardly comparable with natural ecosystems. While the multiple coring approach is sometimes undertaken i.e. to track past water-level changes^47,48^, in such archaeological sites as Rozprza motte, various factors need to be considered, in particular human impact. In waterlogged sites, such as the Serteya Neolithic pile-dwelling, the human–environment relation can be tracked alongside the quantitatively reconstructed climatic background^22,49^. Palaeoecological methods are of great importance while tracking the history of the cities, like Gdańsk^50^ and London^51^ with the use of profiles of wet sediments.

In the majority of archaeological sites, if palaeoecological studies are undertaken, they focus on the surroundings of the excavations, mainly because of the lack of wet organic sediments to take core from (e.g.^52,53^). Another issue is the cost and time, which need to be taken into consideration with any additional core. In our case, the cores of sediments were taken as monoliths directly from the walls of archaeological trenches, and the profiles were relatively short, which was a great convenience. The additional cores, however, were examined only with respect to lithological and geochemical composition, accompanied by chironomid (and ceratopogonid) analysis. With the use of macrofossil analysis, habitat diversity could be even more accurately mapped, which is worth considering in future research.

To sum up, multiple cores are required to get a complete picture of the spatio-temporal changes within the ecosystem. The environmental reconstructions from the deepest part of the moat (RP W3(2)) presented in Kittel et al.^5^ are substantiated here, and the results are largely consistent with the core taken from the same trench (RP W3(4)). However, the sequences from the second moat (RP F2) and the NE part of the main moat (RP W1) significantly complement the reconstruction and help give a better understanding of the functioning of moat ecosystems and motte-and-bailey strongholds.

This study represents the first reconstruction of moat habitats during its functioning that considers spatial variation. It is likely that many similar water bodies could be investigated this way, broadening our knowledge about past societies and ecology of such human-made ecosystems.

## Material and methods

### Fieldwork

The research in Rozprza began with a non-destructive survey carried out in 2013–2015. Methods included analytical field walking, aerial photography, geophysical and geochemical prospecting, as well as thorough geological mapping. A dense network of cores taken with a hand auger resulted in elaboration of detailed cross-sections of the ringfort vicinity. Thanks to this investigation it was possible to localise some archaeological and palaeogeographical features^27,54^. This led to the next extensive, interdisciplinary investigation. This fieldwork was conducted in 2015–2016, with the use of archaeological trenches, geological outcrops and a wide range of palaeoecological studies. They aimed to reconstruct the environmental conditions and settlement history of the mediaeval stronghold at Rozprza^5,16,55^.

The procedure of exposing trenches included removing successive 10-cm layers of sediments, distinguishing stratigraphic units within them, and wet-sifting with a 4 × 4 mm sieve in order to collect archaeological artefacts and ecofacts. All trench walls and collected features were thoroughly documented as orthophotos. The sediments for palaeoecological analyses were collected from the trench walls as monoliths using metal boxes with dimensions of 50 × 10 × 10 cm (Supplementary Fig. S1). Thanks to this method, the undisturbed structure of the sediments was preserved.

The RP W1 profile was collected from the deepest section of the trench 1/2015. This trench, with dimensions 2.5 × 12 m, was exposed in the eastern part of the main moat (Fig. 1C). Wooden vertical posts, associated with numerous fragments of wood, were revealed in the bottom of the moat. The moat was shallow here, reaching up to 50 cm depth. Two cores of sediments were collected from the trench 3/2015 (1.5 × 25 m), situated in the south-western part of the main moat. The RP W3(2) profile was taken from the deepest part of the main moat and RP W3(4) from its shallower part. Trench 3/2015 exposed the very well preserved moat fill, adjoining the outer rampart and the motte mound, allowing for their full cross-section. The RP F2 profile was taken from the thoroughly deepened and purified wall of the drainage ditch, which currently crosses the secondary moat. The deposits were collected from the deepest part of the smaller southern moat (Fig. 1C).

### Digital reconstruction of the main moat relief

The 3D reconstruction model of the bottom of the main moat was prepared within the GIS environment (Qgis, SAGA GIS and PlanlaufTerrain softwares) using point cloud of Airborne Laser Scanning (ALS) already accessible via the Geoportal.gov.pl web service. This was supplemented with results of detailed coring (80 drillings 1 to 2 m apart) of the moat as well as results of excavation of archaeological trenches 1/2015 and 3/2015. Contemporary bare earth points covering the moat in the ALS derived point cloud were replaced by points with the height values of the surface of mineral bedrock indicating the original bottom of the moat. Subsequently, all the points were interpolated to obtain a Digital Elevation Model of the stronghold area with the main moat virtually reconstructed and emptied. This allowed for modelling water circulation and subsequent changes of moat states.

### Geochemical and sedimentological analysis

Detailed geochemical tests covered material from the four cores presented here (133 samples from 4 cores) (Fig. 2). The basic physical and chemical parameters were the following: organic matter content (LOI—loss on ignition), calcium carbonate (CaCO_3_) content (volumetric measurement of CO_2_ from conversion of CaCO_3_ by 10% HCl), biophilic elements such as: total organic carbon (TOC), total nitrogen (TN) and total sulphur (TS) content (combustion method at the Rapid CS cube and VarioMax analyser—Elementar), and reaction (pH in distilled water). All parameters were measured in 2-cm resolution according to the procedure by Tolksdorf et al.^56^. Ash material without organic matter (remaining after LOI) was dissolved with concentrated 65% HNO_3_, 10% HCl and H_2_O_2_ in a Berghof Speedwave microwave mineralizer. Elements with palaeoenvironmental significance (Na, K, Ca, Mg, Fe, Mn, Cu, Zn and Pb) identified in the resulting solution were marked by the atomic absorption spectroscopy (AAS) method with use of Solar Unicam and following procedure after Borówka^37^.

When interpreting the individual elements of the chemical composition and grain size distribution for the mineral fraction for all four cores, the variable character of the sediment accumulation conditions was taken into account^57,58^. Palaeoenvironmental conditions responsible for the sedimentation of the studied deposits were interpreted by determining the quantitative ratios of the elements (such as: Na/K, Fe/Mn and Cu/Zn) with the assumption that the individual lithogeochemical components came from different sources (cf.^49^).

The grain size composition of mineral ash (treated as terrigenous silica) remaining after solution was prepared as in Clift et al.^59^, using a Mastersizer 3000 laser particle-size analyser (Malvern). The grain-size data were stored and processed using GRADISTAT software v. 8.0^60^.

### Chironomidae and Ceratopogonidae analysis

The samples for midge analysis were taken as contiguous 2-cm slices of the sediment from each profile, apart from the RP W3(2) core, from which they were collected with 4 cm resolution. The number of samples analysed in each profile was similar (ranging between 23 in RP W1 and 27 in RP W3(2)), while sample volume varied between 5 and 70 cm^3^.

Chironomidae preparation methods followed Brooks et al.^25^. The sediments were passed through a 63 μm mesh sieve. If head capsule (hc) concentration in the sediments was low, kerosene flotation was used following the procedure of Rolland and Larocque^61^. Processed sediment was scanned under a stereo-binocular microscope. Where applicable, a minimum of 50 (preferably 100) chironomid head capsules from each sample were picked and mounted in Euparal^®^. Identification of chironomids followed Schmid^62^, Brooks et al.^25^, and Andersen et al.^63^, while ceratopogonids were divided into two morphotypes distinguished by Walker^64^. Ecological preferences of identified taxa are based mainly on Brooks et al.^25^, Vallenduuk and Moller Pillot^65^, Moller Pillot^30,66^, and Luoto^31^. The midge sequences are presented on stratigraphic diagrams (Supplementary Figures S3–S6) created with C2 software^67^.

### Radiocarbon and dendrochronological dating

The chronology of the Rozprza moat system was estimated using radiocarbon (^14^C) and dendrochronological methods. Both dendrochronological and conventional radiocarbon dating of organic material using the LSC technique were performed in the Laboratory of Absolute Dating in Kraków (Poland). A few wood fragments sampled during moat system exploration^16^ were dendrochronologically dated using standard procedures^68^.

A total of 15 samples from moats of the Rozprza motte-and-bailey were collected for radiocarbon (^14^C) dating (Table 1). Thirteen of these were sampled from three cores of the main moat and two were from the southern secondary moat (cf.^16^). For the full cross-section of the deepest part of the main moat, seven dates were obtained for the RP W3(2) core and four for RP W3(4)^5^. Two more ^14^C datings were made for the RP W1 core from the eastern shallow part of the main moat, and a further two for the RP F2 core in the southern additional moat.

Twelve samples of bulk organic deposits (organic mud, peat or gyttja) were dated using the liquid scintillation technique (LSC) and three samples of selected terrestrial plant macrofossils dated using the accelerator mass spectrometry technique (AMS). All samples were chemically pre-treated using the AAA (acid–alkali–acid) method. The procedure for conventional radiocarbon dating of organic material using the liquid scintillation counting method (LSC) included the standard synthesis of benzene from organic samples^69^. ^14^C measurements were carried out with a 3-photomultiplier spectrometer, the HIDEX 300SL and Quantulus 1220. Organic samples dated using the AMS technique were combusted, purified, and graphitised with Fe catalyst^70^. The mixture of graphite and Fe powder was pressed into a target holder and measured with the AMS system at the Centre for Applied Isotope Studies at the University of Georgia, USA or in the Accelerator Mass Spectrometry Laboratory (D-AMS laboratory code) in Seattle, USA (see^71^ for details).

Calibrated radiocarbon ages (BC/AD) were made using the IntCal20 radiocarbon calibration dataset^72^ and the OxCal 4.4.2 calibration software^73,74^. The age-depth curves for studied cores were elaborated based on the OxCal P_Sequence model^75^. The age-depth models were obtained separately for four studied cores. More detailed chronology was obtained for the longest RP W3(2) core, and the new model slightly differs from that published by Kittel et al.^5^. A dendrochronological date from a fragment of wooden ecofact found in the very bottom of the main moat was included into RP W3(2) and RP W3(4) age-depth models (cf.^5^). For an estimation of absolute chronology of selected palaeoenvironmental events, the probability distributions of the modelled calendar ages for 1-cm intervals of deposits were calculated (Supplementary Tables S1–S4). These were used for estimation of absolute chronology of selected palaeoenvironmental events.

### Statistical data analyses

#### Self-organising map and indicator species analysis

Patterns in the dipteran assemblages were recognized with Kohonen’s (unsupervised) artificial neural network (ANN), also referred to as a self-organising map (SOM)^76,77^. Artificial neural networks (ANNs) are simple structural and functional models of the brain. ANNs have many advantages, which allow a researcher to apply them to “difficult” data. They do not require any a priori specification of the model underlying a studied phenomenon because they learn it based on the processed data. They are also robust to noise in data^78,79^. This is important for the purposes of the present study, because taxa abundances in field samples do not reflect exactly the original abundances of populations^80^. Additionally, in palaeoecological research the long time separating the living populations and their sampling, and resulting decomposition and fragmentation additionally enhance the problem^81^. ANNs are also robust to non-linear relationships between variables and to non-normal distributions in data^82,83^. This is also crucial in this study because the counts of rare species cannot be effectively normalised by any transformation due to their absence in most samples and therefore strongly skewed variable distributions^82,84^. Furthermore, dipteran assemblages are shaped by many abiotic and biotic factors that are related in complex ways. The SOM application in palaeoecology and its advantage in comparison to classical zonation methods (CONISS and Optimal Partitioning) are well described by Płóciennik et al.^85^.

Kohonen’s ANNs are constructed from data processing units (neurons) arranged in two layers: an input layer used for data input, and an output layer responsible for data structuring and output. The data used for the SOM analysis comprised log-transformed abundances of 97 taxa recorded in 79 non-empty core samples.

They were displayed on the input layer comprising 97 neurons (one input neuron per taxon). The output neurons were arranged as a two-dimensional rectangular lattice. The number of output neurons should be close to 5√*n*, where *n* is the number of samples; in this case the result was 44 (see^86^). Therefore, the final size of the lattice was 7 × 6 (= 42) neurons.

Each input neuron repeatedly transmitted signals to each output neuron. These signals were strengthened or weakened by modifying the weight of the connections between neurons. On this basis, a virtual dipteran core sample (DCS) was created in each output neuron.

The distance between virtual DCSs on the two-dimensional lattice exhibited their mutual dissimilarity, i.e. virtual DCSs in distant output neurons differed considerably while those in neighbouring output neurons were similar. The latter might not be true when the neighbouring output neurons were in different (sub)clusters as the virtual DCSs, and respective output neurons, were additionally clustered with hierarchical cluster analysis (with Ward algorithm and Euclidean distance)^78,86,87^.

Finally, each real DCS was assigned to the best matching virtual DCS and the respective output neuron. Therefore, the mutual distance of the real DCSs on the two-dimensional lattice was a derivative of the mutual dissimilarity and position of virtual DCSs: significantly dissimilar real DCSs were located in distant neurons, while similar real DCSs were located in the same neuron or in adjoining neurons^83^.

The batch training algorithm was chosen for the purpose of network training, because it does not require any training rate factor to be specified^78^. The network training and the clustering of virtual DCSs were performed with the use of the SOM Toolbox^88^ developed by the Laboratory of Information and Computer Science at the Helsinki University of Technology (http://www.cis.hut.fi/projects/somtoolbox/).

The SOM Toolbox allows the associations between dipteran taxa and SOM regions to be visualised in the form of greyness gradients over the two-dimensional lattice^83^. This visualisation may facilitate the formulation of ecological conclusions as taxa with the same patterns of greyness usually co-occurred and exhibited similar habitat preferences.

However, the SOM Toolbox does not provide a statistical verification of those associations. For this reason, the untransformed dipteran abundance data were subjected to Indicator Species Analysis (ISA): the associations between each dipteran taxon and each subcluster of output neurons, and its respective environmental conditions, were expressed in a numeric form with the indicator values (IndVals)^89^. IndVals complement the visualisation in the form of greyness gradients. An IndVal (range 0–100%) of taxon *i* in all real DCSs of subcluster *j* is a product of three values: (1) A_ij_—a measure of specificity, i.e. the mean abundance of taxon *i* in real DCSs assigned to subcluster *j* divided by the sum of its average abundances in all subclusters (%), (2) F_*ij*_—a measure of fidelity, i.e. the frequency of occurrence of taxon *i* (%) in real DCSs assigned to subcluster *j*, and (3) the constant 100 in order to produce the percentages:

$${\text{IndVal}}_{ij} = {\text{A}}_{ij} \times {\text{F}}_{ij} \times {1}00$$

$${\text{A}}_{{{\text{ij}}}} = {\text{ taxon}}\;{\text{abundance}}_{ij} /{\text{ taxon}}\;{\text{abundance}}_{i.}$$

$${\text{F}}_{ij} = {\text{ N}}\;{\text{real}}\;{\text{core}}\;{\text{samples}}_{ij} /{\text{N}}\;{\text{real}}\;{\text{core}}\;{\text{samples}}_{.j}$$

The maximum IndVal (100%) was observed when all real DCSs with taxon *i* were assigned to subcluster *j* and when taxon *i* was present in all real DCSs assigned to subcluster *j*^89^. Significant maximum IndVals, and therefore significant associations of individual taxa with a given SOM subcluster (and its respective environmental conditions), were identified with Monte Carlo randomisation statistics. The significance level was calculated as the proportion of randomised trials with IndVal exceeding or equal to the observed IndVal. The above calculations were performed in PC-ORD^90^.

#### Generalised linear mixed model

We asked whether chironomid taxon composition was consistent across sediment cores, so that results from a single core could be extrapolated across the whole site. Following Hadfield et al.^91^, we used a poisson family generalised linear mixed model (GLMM) with log link to test whether relative species abundances differed between cores RP W1, RP W3(2) and RP W3(4). Core RP F2 was excluded due to low temporal overlap of RP F2 sample ages with other cores, particularly core RP W1 (Fig. 2). Three samples with no age estimate from radiocarbon dating were excluded. Chironomid morphotypes that were absent from a core, age category, or sample were included as zero counts. After reducing samples to only those with strongly overlapping ages among the three cores, sample age was converted to a factor with 6 levels, to allow for nonlinearities in changes in species abundance over time. All resulting age categories were represented by all studied cores. This resulted in a dataset with 5141 individual species counts.

The response variable in the generalised linear mixed model (GLMM) was the untransformed count of individuals of each chironomid morphotype in each sample. To control for variation in sediment volume among samples, we included ln(sample volume cm^3^) as an offset (logged because we fit a poisson model with log link), so that the fixed effect parameter estimates represented the effects of predictors on chironomid counts per unit sediment volume. Fixed effect predictors included core, chironomid taxon (morphospecies), and their interaction. Sample age category and its pairwise interactions with core and morphotype were included as random effects to control for temporal variation in abundances. The core: morphospecies interaction fixed effect therefore tested for differences among cores in relative species abundance, controlling for differences in overall abundance among cores and morphospecies (fixed main effects), sample volume (offset), and any influence of sample age (random effects). All analyses were run in R version 4.1.2^92^. GLMM was performed using the R package glmmTMB^93^. Model diagnostics were performed in DHARMa^94^, and fixed effect model selection based on AICc carried out in MuMIn^95^. All random effects were included in every model.

Post-hoc comparison of relative species abundance differences among cores for individual chironomid morphotypes, based on the estimated core:morphospecies interaction, were carried out in the R package phia^96^. This package is not compatible with mixed effects models and so these analyses are based on a GLM model including fixed effects only, run using base R’s glm function.

#### Canonical correspondence analysis

Because the performed DCA for all four combined cores dataset revealed long biological data gradients (4.499 on Ax 1 and 4.787 on Ax 2 [SD units]), Canonical Correspondence Analysis (CCA) was selected to compare geochemical and biotic variable patterns. Due to autocorrelation, Na and Mg content were excluded from the further analysis. The CCA was performed on square-root transformed data with downweighting rare taxa, biplot scaling and inter-sample distance. The significance of environmental variables relating to the biota was tested with the Monte Carlo permutation with automatic selection and permutation under full model.

## Supplementary Information

Supplementary Information.

## Data Availability

The datasets analysed during the current study are available from the corresponding author on reasonable request.
