# Supplementary material for: The spatial and temporal reconstruction of a medieval moat ecosystem
Source: Sci Rep. 2022 Nov 30;12:20679. doi: 10.1038/s41598-022-24762-w (PMC9712582; doi:10.1038/s41598-022-24762-w)

A closer look into a medieval moat – the temporal and spatial chironomid-based reconstruction of habitat mosaic and ecosystem functioning

Olga Antczak-Orlewska, Daniel Okupny, Andrzej Kruk, Richard Ian Bailey, Mateusz Płóciennik, Jerzy Sikora, Marek Krąpiec, Piotr Kittel

Supplementary information

Supplementary Table S1. The P_Sequence depth/age model for the RP W3(2) core. Indices: A_{model} = 63.1, $A_{overall}$ = 63.7.

Name	Depth (cm b.g.l.)	^{14}C age (yr BP)	Calibrated age (cal AD)		Modelled age (AD)		Mean age ±1σ (AD)	Indices	
			68.2%	95.4%	68.2%	95.4%		A	C
Boundary Top	0.0				1701-1914	1677-2088	1851 ± 119		95.0
R_Date 42-47	44.5	120±40	1690-1922	1647-1943	1673-1856	1667-1919	1771 ± 76	97.1	98.0
R_Date 55-60	57.5	230±35	1641-1800	1525-…	1641-1796	1635-1802	1695 ± 57	114.4	99.1
R_Date 75-80	77.5	320±40	1506-1639	1472-1650	1513-1637	1490-1645	1568 ± 45	105.0	97.6
R_Date 85-90	87.5	370±35	1458-1622	1449-1635	1460-1523	1452-1625	1517 ± 48	108.7	98.1
R_Date 92-94	93.0	409±36	1440-1615	1428-1629	1448-1502	1436-1618	1494 ± 47	104.7	99.2
R_Date 134-136	135.0	715±43	1265-1380	1225-1390	1325-1378	1325-1387	1345 ± 21	22.8	98.0
C_Date 136*	136.0				1328-1329	1328-1329	1329	100.0	100.0
Boundary Bottom	136.0				1328-1329	1328-1329	1329		100.0

OxCal v4.4.2 [73]: r.5 Atmospheric curve [72]
* dendrochronological data of wood fragment 1329 AD

Supplementary Table S2. The P_Sequence depth/age model for the RP W3(4) core. Indices: A_{model} = 98.8, $A_{overall}$ = 98.7.

Name	Depth (cm b.g.l.)	^{14}C age (yr BP)	Calibrated age (cal AD)		Modelled age (AD)		Mean age ±1σ (AD)	Indices	
			68.2%	95.4%	68.2%	95.4%		A	C
Boundary Top	0.0				1717-2051	1689-2348	1969 ± 174		97.6
R_Date 38-43	40.5	100±40	1695-1916	1679-1941	1703-1912	1681-1940	1823 ± 74	100.6	99.3
R_Date 50-55	52.5	200±40	1656-…	1640-…	1647-1796	1529-1846	1713 ± 62	104.3	99.7
R_Date 60-65	62.5	460±40	1420-1459	1401-1615	1420-1465	1404-1620	1460 ± 48	92.8	99.8
C_Date 83*	83.0				1328-1329	1328-1329	1329	100.0	100.0
Boundary Bottom	83.0				1328-1329	1328-1329	1329		100.0

OxCal v4.4.2 [73]: r.5 Atmospheric curve [72]
* dendrochronological data of wood fragment 1329 AD

Supplementary Table S3. The P_Sequence depth/age model for the RP W1 core. Indices: A_{model} = 96.8, $A_{overall}$ = 97.

Name	Depth (cm b.g.l.)	^{14}C age (yr BP)	Calibrated age (cal AD)		Modelled age (AD)		Mean age ±1σ (AD)	Indices	
			68.2%	95.4%	68.2%	95.4%		A	C
Boundary Top	0.0				1427-1570	1413-1878	1554 ± 133		96.8
R_Date 20-22	21.0	479±22	1425-1442	1414-1450	1424-1442	1413-1450	1432 ± 9	98.0	99.9
R_Date 40-45	42.5	610±50	1305-1397	1289-1415	1313-1407	1295-1421	1360 ± 36	97.8	97.7
Boundary Bottom	45.0				1309-1405	1229-1430	1340 ± 72		97.2

OxCal v4.4.2 [73]: r.5 Atmospheric curve [72]

Supplementary Table S4. The P_Sequence depth/age model for the RP F2 core. Indices: A_{model} = 98.5, $A_{overall}$ = 98.5.

Name	Depth (cm b.g.l.)	^{14}C age (yr BP)	Calibrated age (cal AD)		Modelled age (AD)		Mean age ±1σ (AD)	Indices	
			68.2%	95.4%	68.2%	95.4%		A	C
Boundary Top	0.0				1749-2269	1741-2606	2121 ± 239		96.4
R_Date 38-43	40.5	80±40	1695-1916	1683-1936	1688-1909	1674-1927	1792 ± 81	98.1	99.7
R_Date 60-65	62.5	340±50	1484-1634	1455-1646	1501-1641	1459-1650	1567 ± 58	99.8	99.1
Boundary Bottom	65.0				1491-1636	1261-1794	1522 ± 127		98.4

OxCal v4.4.2 [73]: r.5 Atmospheric curve [72]

Supplementary Table S5. List of all recorded Chironomidae and Ceratopogonidae morphotypes with their codes used in CCA analysis. Relative abundance of each taxon in each of the three cores, as estimated by the core:species interaction in a generalised linear mixed model, and controlling for absolute abundance per core and taxon (fixed effects).

Full morphotype name	Morphotype code	Core		
		RP W1	RP W3(2)	RP W3(4)
Chironomus plumosus-type	Chplu	-10.18747	11.15291	-0.96544
Dicrotendipes notatus-type	Dnot	-7.17553	8.50099	-1.32546
Ablabesmyia	Ablab	-4.95403	6.34782	-1.39379
Microtendipes pedellus-type	Miped	-4.42917	2.44485	1.98432
Cricotopus intersectus-type	Crint	-4.01220	7.91084	-3.89864
Procladius	Procl	-3.99015	5.28209	-1.29193
Polypedilum nubeculosum-type	Pnube	-3.49460	1.92962	1.56498
Paratanytarsus penicillatus-type	Parpe	-2.84300	5.49873	-2.65573
Tanytarsus nemorosus-type	Tnemo	-2.58333	5.75840	-3.17508
Endochironomus impar-type	Endim	-2.39718	2.56546	-0.16828
Corynoneura arctica-type	Corar	-2.34443	5.36854	-3.02411
Limnophyes	Limnop	-1.77060	4.02722	-2.25662
Guttipelopia	Guttip	-1.13742	2.63458	-1.49716
Chironomus anthracinus-type	Chant	-0.91131	0.32552	0.58579
Tanytarsus pallidicornis-type 2	Tpal2	-0.86363	0.96176	-0.09813
Phaenopsectra flavipes-type	Phfla	-0.58267	-0.03869	0.62135
Cricotopus bicinctus-type	Crbic	-0.57186	1.60563	-1.03377
Cricotopus laricomalis-type	Crlar	-0.33296	1.21577	-0.88281
Psectrocladius sordidellus-type	Pssor	-0.30111	0.87541	-0.57430
Tanypus	Tanypu	-0.26548	0.77649	-0.51100
Bezzia-type	CBezz	-0.19722	0.94785	-0.75063
Parametriocnemus-Paraphaenocladius	Paraph	-0.16687	0.79210	-0.62523
Cricotopus cylindraceus-type	Crcyl	-0.14776	-0.24509	0.39285
Endochironomus tendens-type	Endte	-0.12772	0.21006	-0.08234
Cladopelma goetghebueri-type	Clgoe	-0.12254	-0.38209	0.50463
Monopelopia tenuicalcar	Monot	-0.00378	0.73764	-0.73386
Polypedilum sordens-type	Psord	0.02056	0.33067	-0.35123
Glyptotendipes pallens-type	Glpal	0.04143	-0.16657	0.12514

Tanytarsus pallidicornis-type 1	Tpal1	0.05388	0.56263	-0.61651
Paramerina-*Zavrelimyia*	ParZv	0.14617	-0.81379	0.66761
Dicrotendipes nervosus-type	Dnerv	0.14643	-0.08923	-0.05719
Tanytarsus mendax-type	Tmend	0.26586	0.00253	-0.26838
Corynoneura edwardsi-type	Cored	0.27611	-0.03498	-0.24113
Zavreliella	Zavrla	0.30095	0.01752	-0.31847
Psectrocladius limbatellus-type	Pslim	0.31852	-0.26168	-0.05684
Einfeldia pagana-type	Epag	0.39034	-2.13646	1.74612
Corynoneura coronata-type	Corco	0.39065	-0.18955	-0.20110
Parachironomus varus-type	Parva	0.43068	-0.50162	0.07094
Cladotanytarsus mancus-type	Cladm	0.49879	-0.40584	-0.09296
Psectrocladius barbatipes-type	Psbar	0.55727	-0.72713	0.16986
Psectrotanypus varius	Psvar	0.57131	-0.52320	-0.04811
Chaetocladius piger-type	Chpig	0.59377	-0.55607	-0.03770
Acricotopus	Acric	0.60384	-0.90189	0.29805
Tanytarsus lactescens-type	Tlact	0.60694	-0.62213	0.01519
Glyptotendipes caulicola-type	Glcau	0.66294	-1.67776	1.01482
Glyptotendipes barbipes-type	Glbar	0.68749	-0.78679	0.09930
Cricotopus tremulus-type	Crtre	0.71508	-0.83842	0.12333
Cricotopus obnixus-type	Crobn	0.73605	-0.68668	-0.04937
Phaenopsectra type A	PhA	0.74107	-1.35441	0.61333
Paratanytarsus type A	ParA	0.75473	-0.96099	0.20626
Psectrocladius flavus-type	Psfla	0.75913	-1.17413	0.41500
Paratendipes albimanus-type	Paral	0.76403	-0.92781	0.16378
Lauterborniella	Lauter	0.80870	-1.71810	0.90941
Pseudosmittia	Psmitt	0.82449	-0.97423	0.14974
Cryptochironomus	Crypch	0.86413	-1.09680	0.23266
Micropsectra pallidula-type	Mpall	0.87730	-1.16285	0.28555
Nanocladius rectinervis-type	Nanre	0.87730	-1.16285	0.28555
Paracricotopus	Paracr	0.87730	-1.16285	0.28555
Rheotanytarsus	Rheota	0.87730	-1.16285	0.28555
Diplocladius	Diploc	0.88132	-1.18650	0.30518
Kiefferulus tendipediformis	Ktend	0.88446	-1.48012	0.59566
Thienemannimyia group	Thiene	0.89574	-1.17207	0.27633
Paratanytarsus austriacus-type	Parau	0.89889	-1.46570	0.56681

Glyptotendipes severini-type	Glsev	0.91695	-1.28542	0.36847
Tanytarsus chinyensis-type	Tchin	0.91695	-1.28542	0.36847
Dasyhelea-type	CDasy	0.92774	-1.43684	0.50910
Natarsia	Natars	0.93137	-1.27099	0.33962
Pseudorthocladius	Psorth	0.93137	-1.27099	0.33962
Endochironomus albipennis-type	Endal	0.93665	-1.21416	0.27751
Smittia foliacea-type	Smitf	0.97078	-1.12848	0.15770
Einfeldia natchitocheae-type	Enat	0.97102	-1.39357	0.42255
Cladopelma viridulum-type	Clvir	0.98544	-1.37914	0.39369
Hydrobaenus conformis-type	Hydco	0.98544	-1.37914	0.39369
Micropsectra contracta-type	Mcont	0.98544	-1.37914	0.39369
Rheocricotopus fuscipes-type	Rheof	0.98544	-1.37914	0.39369
Stictochironomus rosenshoeldi-type	Stros	0.98544	-1.37914	0.39369
Tanytarsus glabrescens-type	Tglab	0.98544	-1.37914	0.39369
Tanytarsus lugens-type	Tluge	0.99624	-1.53056	0.53432
Corynoneura antennalis-type	Coran	1.00389	-1.38836	0.38447
Micropsectra insignilobus-type	Mins	1.01066	-1.51614	0.50547
Paracladopelma	Paracl	1.01066	-1.51614	0.50547
Polypedilum convictum-type	Pconv	1.01066	-1.51614	0.50547
Eukieferiella devonica-type	Eudev	1.02509	-1.50171	0.47662
Stenochironomus	Stenoc	1.02509	-1.50171	0.47662
Eukiefferiella	Eukief	1.03952	-1.48728	0.44777
Metriocnemus eurynotus-type	Meteu	1.03952	-1.48728	0.44777
Micropsectra junci-type	Mjunc	1.03952	-1.48728	0.44777
Stempellinella-Zavrelia	StZav	1.04078	-1.40681	0.36603
Einfeldia dissidens-type	Ediss	1.04353	-1.51093	0.46740
Anatopynia	Anatop	1.05796	-1.49651	0.43854
Cryptotendipes	Crypte	1.05796	-1.49651	0.43854
Metriocnemus terrester-type	Mette	1.05796	-1.49651	0.43854
Stempellina	Stempe	1.05796	-1.49651	0.43854
Clinotanypus nervosus	Clinn	1.06198	-1.52015	0.45818
Brillia flavifrons-type	Brilf	1.07640	-1.50573	0.42932
Neozavrelia	Neozav	1.07640	-1.50573	0.42932
Polypedilum nubifer-type	Pnubi	1.16578	-1.10704	-0.05874

Supplementary Figure S1. The trench walls in the moment of collecting monoliths of the secondary (A) and main moat (B-D) deposits. Photos show the sediment layers and the fragments of wood.

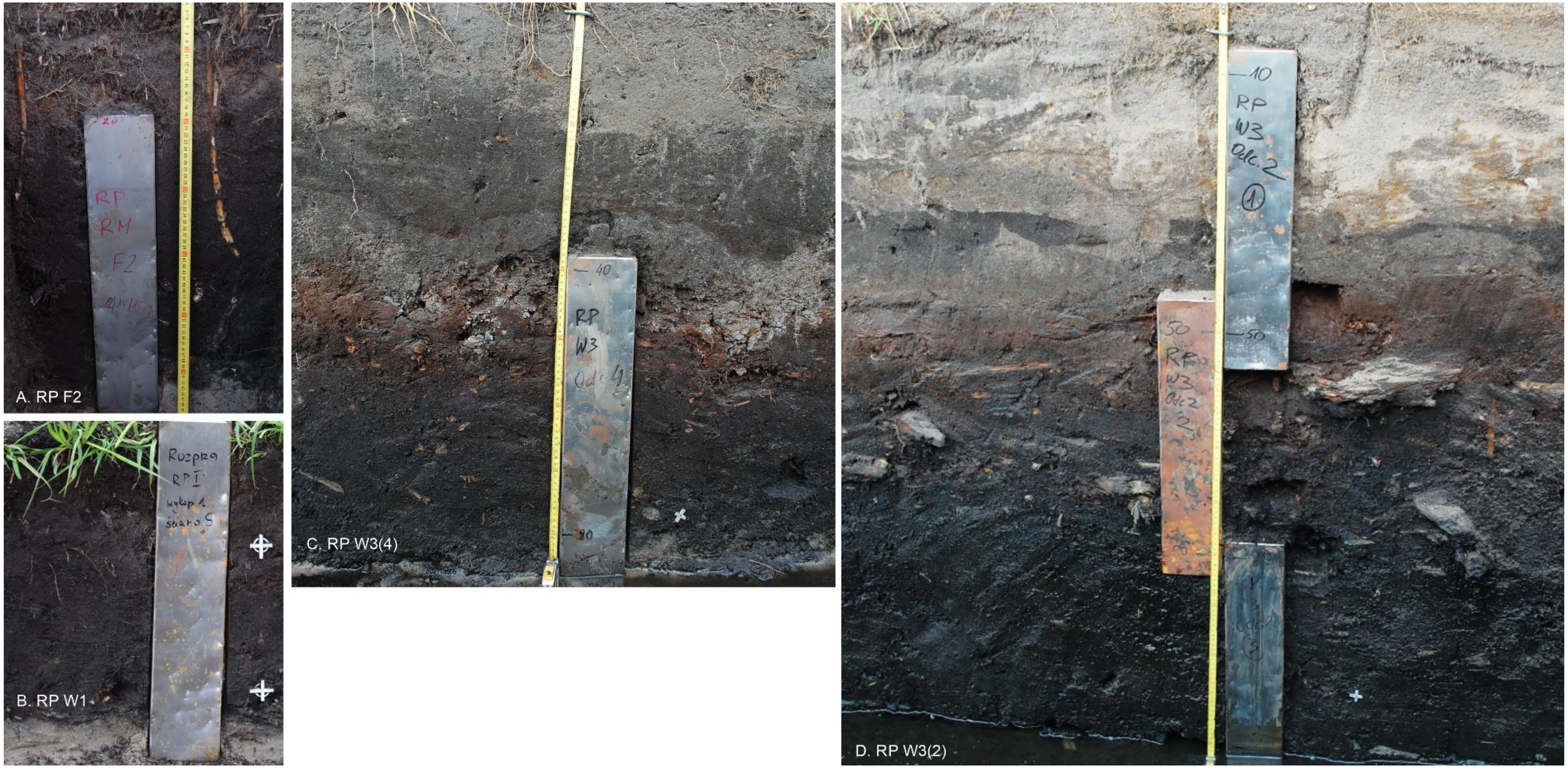

Supplementary Figure S2. Relationship between mean grain size (Mz) and sorting (δ1) for mineral fraction from three cores from Rozprza site.

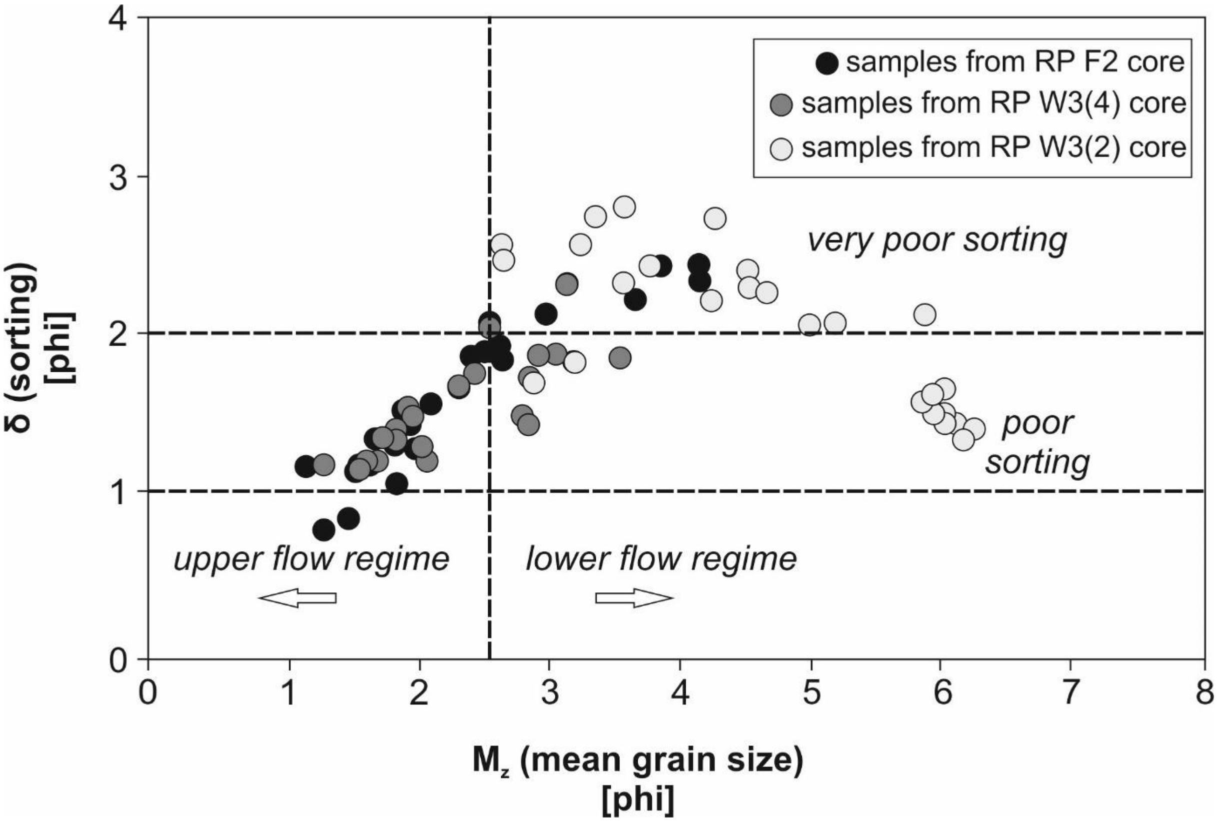

Supplementary Figure S3. Percentage Chironomidae and Ceratopogonidae stratigraphic diagram for the main moat fill from core RP W3(2). The graphs of taxa indicative for each SOM subcluster (see **Fig. 3**) are coloured respectively. The exaggeration curves (multiplier=3) are shown to better present even the lowest shares.

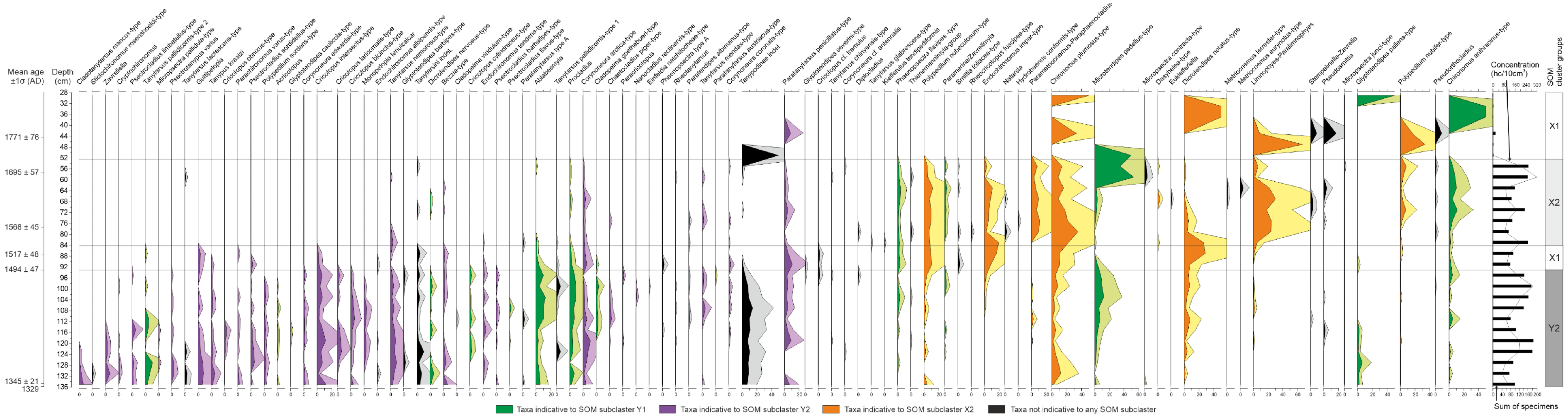

Supplementary Figure S4. Percentage Chironomidae and Ceratopogonidae stratigraphic diagram for the main moat fill from core RP W3(4). The graphs of taxa indicative for each SOM subcluster (see **Fig. 3**) are coloured respectively. The exaggeration curves (multiplier=3) are shown to better present even the lowest shares.

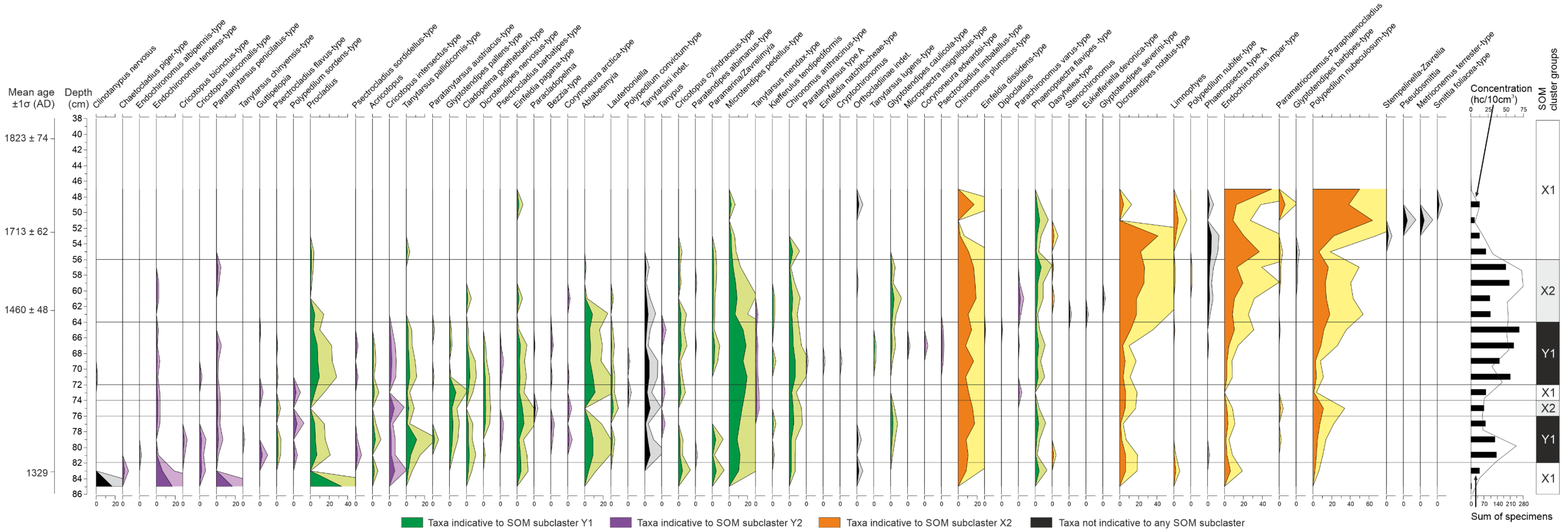

Supplementary Figure S5. Percentage Chironomidae and Ceratopogonidae stratigraphic diagram for the main moat fill from core RP W1. The graphs of taxa indicative for each SOM subcluster (see **Fig. 3**) are coloured respectively. The exaggeration curves (multiplier=3) are shown to better present even the lowest shares.

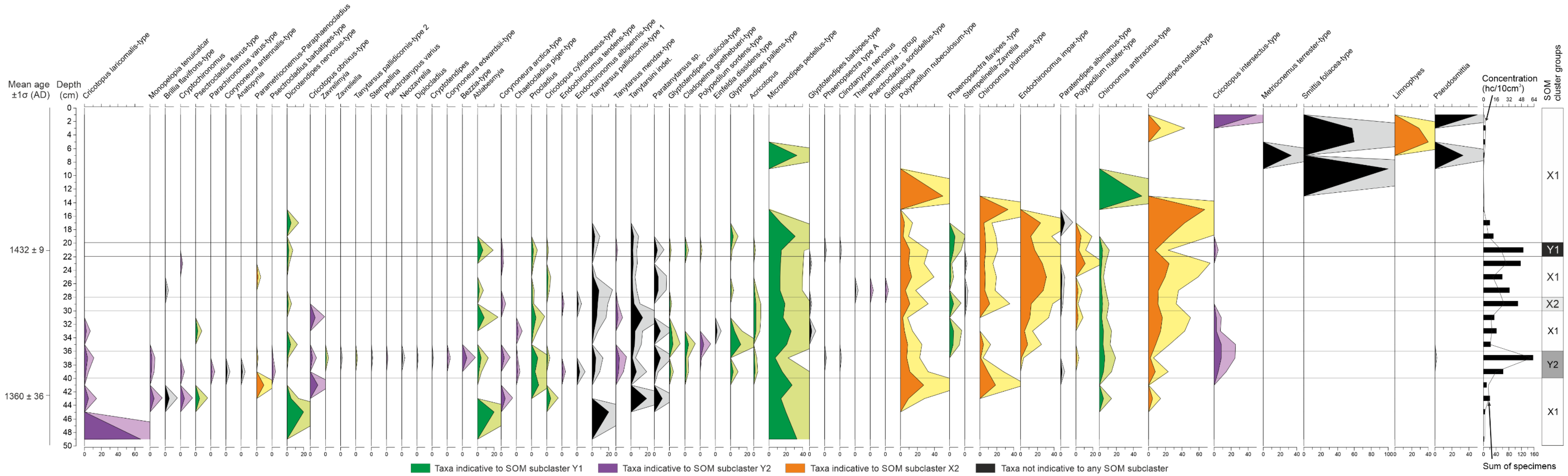

Supplementary Figure S6. Percentage Chironomidae stratigraphic diagram for the secondary moat fill from core RP F2. The graphs of taxa indicative for each SOM subcluster (see **Fig. 3**) are coloured respectively. The exaggeration curves (multiplier=3) are shown to better present even the lowest shares.

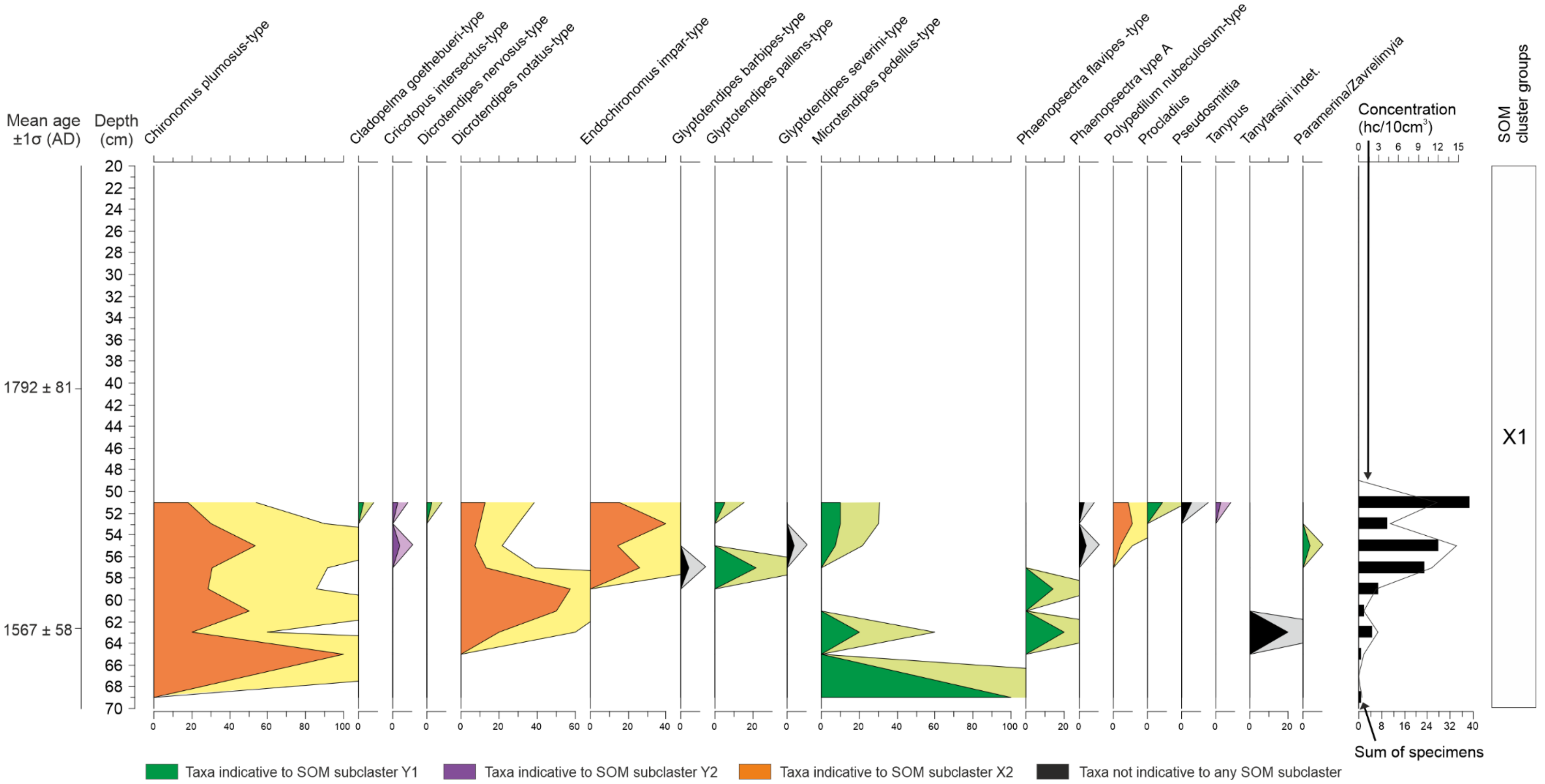

Supplement: Supplementary file 1 — Supplementary Information. [file 41598_2022_24762_MOESM1_ESM.pdf]
